# Medium Access Control Protocols for the Internet of Things Based on Unmanned Aerial Vehicles: A Comparative Survey

**DOI:** 10.3390/s20195586

**Published:** 2020-09-29

**Authors:** Shreya Khisa, Sangman Moh

**Affiliations:** Department of Computer Engineering, Chosun University, 309 Pilmun-daero, Dong-gu, Gwangju 61452, Korea; shreya.khisa21@chosun.kr

**Keywords:** internet of things, unmanned aerial vehicle, medium access control, energy efficiency, throughput

## Abstract

The Internet of Things (IoT), which consists of a large number of small low-cost devices, has become a leading solution for smart cities, smart agriculture, smart buildings, smart grids, e-healthcare, etc. Integrating unmanned aerial vehicles (UAVs) with IoT can result in an airborne UAV-based IoT (UIoT) system and facilitate various value-added services from sky to ground. In addition to wireless sensors, various kinds of IoT devices are connected in UIoT, making the network more heterogeneous. In a UIoT system, for achieving high throughput in an energy-efficient manner, it is crucial to design an efficient medium access control (MAC) protocol because the MAC layer is responsible for coordinating access among the IoT devices in the shared wireless medium. Thus, various MAC protocols with different objectives have been reported for UIoT. However, to the best of the authors’ knowledge, no survey had been performed so far that dedicatedly covers MAC protocols for UIoT. Hence, in this study, state-of-the-art MAC protocols for UIoT are investigated. First, the communication architecture and important design considerations of MAC protocols for UIoT are examined. Subsequently, different MAC protocols for UIoT are classified, reviewed, and discussed with regard to the main ideas, innovative features, advantages, limitations, application domains, and potential future improvements. The reviewed MAC protocols are qualitatively compared with regard to various operational characteristics and system parameters. Additionally, important open research issues and challenges with recommended solutions are summarized and discussed.

## 1. Introduction

Internet of Things (IoT) applications have become increasingly popular in various civil and industrial domains owing to their easy integration with wireless-sensor networks, cost-effectiveness, easy deployment, low energy consumption, etc. In an IoT system, thousands of devices can be connected to each other as well as to the Internet for sharing information. IoT is widely utilized in fields such as healthcare monitoring [1], environmental monitoring [2], remote patient monitoring [3], precision agriculture [4], energy monitoring [5], indoor monitoring [6], dam monitoring [7] and transportation. Moreover, IoT is fundamental for building smart cities [8], whose building blocks include smart metering, smart building, smart agriculture, smart homes, and smart health. The IoT devices can be controlled remotely to perform the desired functionality. The information exchange among the IoT devices occurs through the network that employs the standard communication protocols.

Recently, unmanned aerial vehicles (UAVs) have been used in various military and civil applications. UAVs are lightweight aircraft that can be operated remotely or in a preprogrammed manner. Generally, a UAV is equipped with various sensors, computational units, cameras, a global positioning system, transceivers, etc. UAVs have practical applications in surveillance and monitoring [9], precision agriculture [10], search and rescue operations [11], road traffic monitoring [12]. Conventional communication systems focus on the infrastructure-based networks (such as cell-tower-based LTE and access-point-based Wi-Fi) that have spread to every corner. However, the lack of mobility hinders their adaptation to dynamic mobile environments. Moreover, their high cost and comprehensive deployment procedure render them unsuitable for remote and emergency situations. Therefore, nonconventional communication systems, such as Project Loon [13] and Facebook drone project [14], have appeared. Small-scale UAVs have gained more popularity for more dynamic and ad-hoc scenarios owing to their maneuverability, ease of deployment, hovering ability, and cost-effectiveness.

The integration of UAVs with IoT networks is a new direction for research and industry. The concept of IoT enables things to be connected anywhere anytime using any network, to provide any service. This characteristic feature of IoT allows UAVs to become an integral part of IoT infrastructure. In UAV-based IoT (UIoT), UAVs can be utilized for different purposes, such as UAV trajectory planning [15], data collection from ground IoT devices [16], data sampling and reconstruction [17], energy-efficient device discovery [18]. The usage of drones can enhance the various aspects of smart cities, such as data collection, privacy and security, public safety, disaster management, energy consumption, and quality of life. In UIoT, UAVs generally collect data from ground sensors and devices through peer-to-peer connections. Therefore, data transmission to neighboring nodes is not required, which can reduce energy consumption.

In UIoT, a medium access control (MAC) protocol is essential because it manages the coordination among different IoT devices during data transmission. However, several challenges need to be addressed at the MAC layer to provide high network throughput, low energy consumption, and low latency. The high mobility of UAVs is one of the most important challenges, resulting in a highly dynamic network topology. The IoT devices can only get access to a UAV when the UAV is within their communication range. The UAV is usually equipped with directional antennas for energy-efficient transmission. In this case, the IoT devices located in different areas can communicate with the UAV at different times. This causes unfair access opportunities in the network. On the other hand, reducing the energy consumption of the devices is very important in the UIoT network. In UIoT, three types of energy consumption should be considered: the energy consumption of battery-powered UAVs, energy consumption of onboard sensors, cameras, and other IoT devices, and energy consumption of ground IoT devices and sensor nodes. Therefore, how to select or design an appropriate MAC protocol for the uplink channel by handling these issues is a challenging problem.

The UIoT is a relatively new research area but can solve well-known IoT problems such as data collections from an infrastructure-less remote area, non-line-of-sight (NLoS) communication, energy wastage due to long-distance transmission, and providing network coverage to disaster areas. The main purpose of this article is to summarize the existing MAC solutions in UIoT, which will work as a starting point for researchers and engineers in this area. The rigorous comparison and discussion will help them to get a good insight into existing works in this field.

To the best of the authors’ knowledge, this is the first survey on MAC protocols for UIoT. We surveyed state-of-the-art MAC protocols and compared them with regard to their major features, operational characteristics, and performance metrics. The comparative discussion is beneficial to readers who wish to use the existing protocol or develop a new one for a specific application. The main contributions of this study are as follows:

- We present and discuss the communication architecture of UIoT, along with common application scenarios and their associated challenges. In addition, we preview the MAC protocols for IoT and UAV networks separately, because the heterogeneous architecture of UIoT consists of both IoT and UAV networks.
- By exploiting the important design considerations of MAC protocols for UIoT, we present a new taxonomy of existing MAC protocols tailored to UIoT based on MAC strategies used to access the channel for data transmission. Hence, the existing MAC protocols for UIoT are classified into three categories. Subsequently, they are reviewed and discussed with regard to the main ideas, operational principles, advantages, limitations, application domains, and future improvements.
- The major features, operational characteristics, and performance metrics of the reviewed MAC protocols are qualitatively compared.
- Finally, important open research issues and challenges that must be overcome for developing a better MAC protocol for UIoT are summarized and discussed. Recommended solutions for each of the open research issues are also provided.

The remainder of this paper is organized as follows. In Section 2, the communication architecture of UIoT and application scenarios with their associated challenges are presented. In Section 3, MAC protocols for IoT and UAVs are summarized and discussed as preliminaries. In Section 4, the design considerations of MAC protocols for UIoT are investigated. In Section 5, the existing MAC protocols for UIoT are classified, reviewed, and discussed with regard to the design concepts, key features, advantages, and disadvantages. In Section 6, the MAC protocols are qualitatively compared. In Section 7, important open issues and research challenges with recommended solutions are discussed. Section 8 concludes the paper.

## 2. UIoT Communication Architecture and Application Scenarios

In this section, we discuss the communication architecture of UIoT, as well as some application scenarios and their associated challenges. The UIoT communication architecture determines how information flows between UAVs, between UAVs and IoT devices, and from UAVs to the ground control center.

### 2.1. Communication Architecture of UIoT

The traditional IoT communication architecture is centralized and highly dependent on the infrastructure (e.g., ground control station, satellite). It is primarily implemented in a star topology such that the communication between IoT devices is facilitated by the infrastructure. The most significant problem with this approach is that the infrastructure becomes the network’s single point of failure; hence, the communication architecture is not fault-tolerant. Moreover, most IoT applications are deployed in remote areas without infrastructure, where no wireless or cellular coverage is available. Most IoT devices are battery-powered and irreplaceable. Transmitting the data to a more distant terrestrial base station (BS) requires more energy. Moreover, signal shadowing and fading occurs owing to blockage and obstacles in the ground environment. In UIoT, these problems are solved by using UAVs to inexpensively collect data from the remotely deployed IoT devices. UAVs have inherent characteristics of flexibility, mobility, and line-of-sight (LoS) communication. Because of the LoS communication ability, UAVs can collect data from IoT devices in an energy-efficient and reliable manner. UAVs have proven to be effective for not only collecting data from remote environments but also providing wireless communication to disaster-affected areas. In a disaster-affected area where the BSs have been destroyed, UAVs provide connectivity to the ground users by passing messages from a distant BS to the affected area [19]. The factors that influence the communication architecture of UIoT networks include the number of UAVs and the use of satellites. Therefore, we classify UIoT communication architectures into single-UIoT architecture, multi-UIoT architecture, space–air–ground integrated network (SAGIN), and UAV equipped with IoT devices.

#### 2.1.1. Single-UIoT Architecture

**Figure 1:**
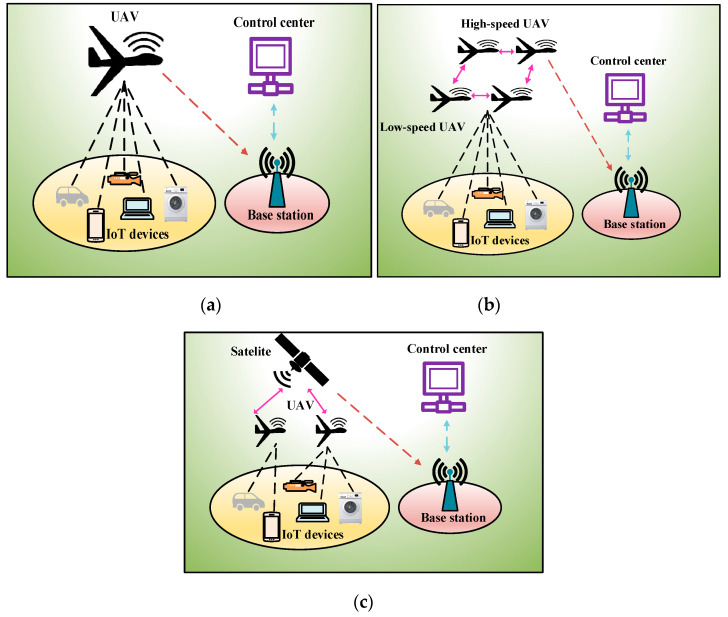
(**a**) Single unmanned aerial vehicle-based internet of things (UIoT) based communication architecture; (**b**) multi-UIoT and multilayer communication architecture; (**c**) space–air–ground integrated network (SAGIN).

This communication architecture is the most suitable for relatively small-scale IoT networks. In Figure 1a, the single-UIoT communication architecture is presented. It is shown in Figure 1a that a single UAV flies to the designated area to collect data from the ground IoT devices. The UAV is rechargeable and must cover the entire area targeted for data collection. The major disadvantage of this architecture is that if the number of IoT devices is large, the UAV energy will be depleted before all the data are collected. In such an architecture, the UAV acts as a communication relay between the ground control station and the remote IoT devices [20].

#### 2.1.2. Multi-UIoT Architecture

This communication architecture is suitable for large IoT networks and IoT networks that generate data frequently. Because of the limited amounts of resources and energy, a single UAV cannot handle all the IoT applications. Therefore, in multi-UAV deployment, the load is shared among the UAVs for data collection. Multi-UAV deployment can be applied in two ways: single-layer and multilayer. In single-layer communication, a group of UAV flies to the target area and collects data. After collecting the data, they return together to the control station. In contrast, in multilayer communication like Figure 1b, the UAVs are deployed in two or more layers. The lower-level UAVs fly at a relatively low speed, and the upper-level UAVs fly significantly faster. The lower-level UAVs collect data and pass them to the upper-layer UAVs. The upper-layer UAVs function as relays, flying back to the ground station [21]. 

#### 2.1.3. SAGIN

SAGINs have recently become popular among researchers and in the industry owing to their flexibility and reliability [22]. Figure 1c represents a SAGIN communication architecture, one or more low-Earth orbit (LEO) satellites are deployed in space, one or more UAVs are deployed in the air, and numerous IoT devices are deployed on the ground. In a SAGIN, the UAV flies close to the IoT devices and collects data. After the data are collected, they are transmitted to the satellite. The satellite is responsible for communicating with the ground stations. Hence, it transmits the data to the ground control stations. This type of communication architecture is suitable for latency-critical applications, as the UAV does not need to fly back to the ground station immediately to transmit its data, which is time-consuming. Instead, it can transfer the data to the satellite. The communication time between the satellite and the ground station is negligible. Therefore, this architecture is suitable for real-time and mission-critical applications.

#### 2.1.4. UAV Equipped with IoT Devices

In this communication architecture, ground IoT devices are not required because the UAV carries the IoT devices and thus collects the data from the environment [23]. However, the load and weight of the UAV are significantly increased, hampering the movement of the UAV. This type of architecture is suitable for air-pollution monitoring in smart cities.

### 2.2. Application Scenarios

#### 2.2.1. UAV-Based Wireless Networks for IoT Devices

The use of UAVs as a flying BS is attracting considerable attention among researchers and in the industry [24]. UAVs can function as a mobile aerial BS to provide reliable downlink and uplink communications for ground users and enhance the capacity of wireless networks. With LoS communication, the UAV can establish strong communication links with the ground devices by mitigating the signal blockage and shadowing. By adjusting its altitude, and speed, the UAV can fly toward potential ground users and establish a reliable connection with low energy consumption. Owing to the energy limitations of IoT devices, they cannot transmit data over long distances. In such IoT scenarios, a UAV can function as a flying BS, collecting data and transmitting it to the devices that are outside of the communication range of the transmitters. In a previous study [25], the energy consumption of IoT devices was significantly reduced via the optimized deployment of multiple UAVs as flying BSs compared with a case in which stationary aerial BSs were deployed. Another study is focused on minimizing the distance between UIoT devices while keeping the UAV connected to the terrestrial base station [26]. Moreover, a technique to optimize the deployment of UAV and user association is addressed in [27].

Associated challenges: One of the design challenges regarding the UAV-based wireless networks is to model air-to-ground (A2G) channels. Compared to air-to-air communications, A2G channels are more prone to signal blockage. Therefore, the optimal design and deployment of UAV-based communication systems require an accurate A2G channel model.

#### 2.2.2. UAVs as Relays for Data Collection from IoT Devices

Recently, the use of UAVs to gather data from IoT devices has attracted research attention. In many IoT applications, IoT devices are deployed in a remote and rural area without ground network infrastructure. These devices produce important data that must be collected periodically. Timely delivery of fresh information is a critical step for data-analytics applications. Because UAVs can provide LoS communication, it is possible to collect data energy efficiently. In the aforementioned situation, a UAV can act as a relay node by collecting data from remote IoT devices and delivering the data to the destination. Many studies have been performed on data-gathering techniques based on UAVs. Researchers mainly focused on optimized path planning for data acquisition [28,29], reducing the energy consumption of IoT devices [30], energy-efficient data collection [31].

Associated challenges: Although UAV’s mobility provides promising opportunities, the trajectory of UAVs needs to be optimized for faster data collection, better throughput, less energy consumption, and lower delay. Generally, optimizing the flight path of UAVs is challenging because it should consider many physical constraints and parameters such as channel variation due to the mobility, UAV’s dynamics, the energy consumption of UAVs, and flight constraints.

#### 2.2.3. UAVs in 5G Communication for IoT Devices

The UAV is an important component of the fifth generation mobile network (5G) and beyond 5G (B5G) communication because of its capability of flexible deployment, strong LoS communication links, and freedom with controlled mobility [32]. 5G must support a larger number of users/devices requiring internet connectivity with different performance requirements and a larger number of applications and use cases. In many situations, terrestrial BSs are inadequate with regard to the 5G key performance indicators because the BS of the cellular network always remains powered. For example, the terrestrial infrastructure may be unable to cover certain areas, such as oceans and rural and remote areas. Most importantly, it may have limited coverage when a disaster occurs and terrestrial BSs become inoperative. In a 5G network, ground IoT devices can increase the available bandwidth that UAVs and satellites can provide. In such scenarios, UAV-based communication is promising for regions outside the coverage of operational ground BSs. The authors of [19] considered disaster scenarios where all BSs are rendered inoperative. To deliver emergency messages from operational BSs outside the disaster region, UAVs are deployed hierarchically. On the other hand, the authors in [33] proposed a hybrid algorithm to minimize the number of UAVs and maximizing the load balancing among the UAVs.

Associated challenges: Due to size, power, and weight constraints, different types of UAVs may be limited to different operational altitudes. The higher altitude of UAVs promotes higher LoS connectivity because reflection and shadowing are reduced, whereas lower altitude ensures a reduction in path loss. In 5G communications, different urban or rural scenarios require different altitudes of UAVs. Thus, optimizing the UAV altitude according to the requirements is a challenging task due to different blockages and obstacles.

#### 2.2.4. UAVs as Energy Harvester for IoT Devices

UAVs can not only collect data but can also wirelessly transfer energy to energy-constrained IoT devices [34]. Most UAVs are rechargeable and can store more energy than an IoT device. Most IoT devices are very small and have a low battery capacity. Thus, UAVs can transfer energy to IoT devices via wireless power transfer (WPT) technology, which can increase the network lifetime.

Associated challenges: As UAVs mainly rely on rechargeable battery power, energy harvesting, and flying duration affect the energy consumption of UAVs significantly. Therefore, it is crucial to prolong the service duration of UAVs during the mission via advanced charging technologies.

#### 2.2.5. UAVs in Mobile Edge Computing (MEC) for IoT Devices

Recently, the use of UAVs in conjunction with MEC has become a popular research topic [35]. Most IoT devices use several IoT applications and must perform several computational tasks. Because of their limited computation capability and energy, IoT devices offload their tasks to a nearby UAV, which is equipped with an MEC server. The UAV performs some of the tasks by itself and offloads the most critical portion to the ground station. After the tasks are completed, the results are transmitted from the UAV to the ground IoT devices.

Associated challenges: Due to the limited computation capability of UAVs, to handle complex offloaded tasks is difficult and also wastes a lot of UAV energy.

#### 2.2.6. UAV-IoT in Crowd Surveillance Using IoT Devices

In general, UAVs exhibit outstanding characteristics compared to manned airplanes. Using suitable IoT devices, cameras, and communication devices, countless use cases can be defined for UAVs. For example, using high-resolution cameras and a suitable communication system such as LTE (Long Term Evolution), UAVs can be used for crowd surveillance [36]. This use can obviously be applicable for security reasons to monitor any suspicious activity among crowds of people. In traditional patrol systems, there is a need for many security guards and a huge amount of human effort to provide the necessary safety for people. In this aspect, UAVs can be used to assist security guards by remotely surveilling people at places of interest. UAVs can also help to track, detect, and recognize criminals adopting face recognition methods.

Associated challenges: UAV-IoT in crowed surveillance may face malicious attacks due to the open links and dynamic topologies by intentional jamming and disruption. To avoid malicious modification, there is a need for a secure and lightweight mechanism to prevent attacks such as eavesdropping, man-in-the-middle attack, and so on.

## 3. Preliminaries

UIoT is a new communication architecture, which comprises both IoT and UAV networks. This section provides the basic knowledge needed to understand the MAC protocols for UIoT. That is, MAC protocols for IoT and UAV networks are briefly reviewed and their characteristics are discussed because of the following motivations: First, the IoT networks solely focus on communication between IoT devices and the control center, and the UAV networks target on communication among UAVs. On the other hand, the UIoT networks emphasize communication between IoT devices and UAV. Second, the issues and challenges related to the MAC protocols for IoT and UAV networks (such as collisions, mobility, and energy wastage) also exist in the UIoT networks. Finally, to find the research gap, it can be necessary to perform a background study of the MAC protocols for UAV and IoT networks separately.

### 3.1. MAC Protocols for IoT

Several MAC protocols have developed to address the challenges of IoT such as energy consumption, low delay, and collision avoidance. An energy-efficient MAC protocol is necessary to increase the network lifetime as most of the IoT devices are battery-powered and remotely located. To handle the energy consumption, the sleep scheduling of IoT devices is a promising technique. A partially synchronous MAC protocol was presented in BirdMAC to reduce energy consumption [37]. In BirdMAC, nodes are allowed to “wake up” only according to an assigned wake-up schedule, and the last node to wake up initiates the communication process. In this protocol, an IoT system sends reports in a quasi-periodic manner. Furthermore, BirdMAC balances the synchronization and coordination cost. However, message exchange for the synchronization and transmission of beacon signals for coordination results in a protocol overhead. BirdMAC offers better energy-saving capabilities with infrequent synchronization of nodes’ clocks. A time-division multiple access (TDMA) and carrier-sense multiple access with collision avoidance (CSMA/CA)-based hybrid MAC protocol targeted for highly dense IoT networks was presented in [38]. It utilizes the energy of the nodes and dynamically adapts the sleep/wake-up period according to the variance in the network loads. The protocol improves the network throughput and energy efficiency by utilizing a dynamic sleep–wake-up method. On the other hand, a receiver-initiated asynchronously duty-cycled protocol was proposed in [39]. However, in the case of UIoT networks, a UAV works as a wireless relay or a BS and collects data from IoT devices. Therefore, all the IoT devices should synchronize their sleep scheduling according to the UAV’s arrival and departure.

Mission- and time-critical application is one of the challenging application scenarios in IoT. This type of application requires low latency as well as high throughput. To tackle this problem, some research works have been conducted. A hybrid scheduling-based MAC protocol called “MAC on time” (MoT) was introduced to ensure the delivery of all uplink packets for mission-critical IoT applications [40]. It improves the utilization of the bandwidth capacity and provides deterministic latency, enhancing the throughput. However, the energy efficiency of the model is not considered in MoT. An efficient prioritized MAC protocol for mission-critical IoT applications was proposed in [41]. This protocol utilizes a random clear access assignment (CCA)-based channel-access mechanism to handle collisions between data packets. It provides high throughput and latency. 

The usage of a multichannel mechanism provides better channel utilization and bandwidth. The authors of [42] presented a traffic-adaptive multichannel MAC protocol based on the 16 channels of IEEE 802.15.4. It adopts phase-lock and dynamic slot allocation schemes to provide low-power duty-cycled communications and increase throughput. However, many other research works have been conducted to solve the MAC layer issues. Ye et al. [43] presented a token-based adaptive MAC (TA-MAC) protocol for a two-hop IoT-enabled MANET. They adopted a TDMA-based superframe structure to overcome the hidden node problem. TA-MAC achieves the minimum average end-to-end delay, a bounded delay for local transmissions, and a high aggregate throughput by utilizing a probabilistic token-passing scheme. Kim et al. [44] proposed an enhanced scheme that addresses the configuration of slot frames, link set slots, enhanced beacon management, and Ipv6 scheduling information for IEEE 802.15.4e time-slotted channel hopping (TSCH) in a large-scale network. In [45], a double-slotted ALOHA-based nonorthogonal multiple access (NOMA)-based MAC protocol for 5G IoT applications was introduced, in which a full throughput efficiency was adopted in cases of low, medium, and high network traffic.

### 3.2. MAC Protocols for UAV Networks

A UAV is a dynamic device that affects the network topology. Because of their high mobility and dynamic topology, UAVs face various challenges in the development of a new MAC protocol such as link quality fluctuations, link failure, and packet collisions. Latency is another challenge when UAVs are deployed for collecting information in disaster and emergency situations. Moreover, due to the dynamicity of the UAV network, the adaptability of the MAC protocol is also desirable. Several studies have been performed to address these challenges of MAC protocols related to UAVs. According to the requirement and use of directional antennas to increase throughput, the MAC switching scheme is an explored field of research. Many research works published considering the issues. An adaptive MAC protocol with fault-tolerant synchronous switching (FS-MAC) for flying ad-hoc networks (FANET) was presented in [46]. It improves the flexibility and robustness of FANETs by utilizing a Q-learning-based MAC switching scheme. FS-MAC exhibited high performance with regard to throughput, delay, and packet retransmission. Moreover, the authors in [47] presented an adaptive MAC called CT-MAC which allows multiple MAC protocols to switch manually based on specific network conditions such as queue length, bit-error frames, and traffic load. A multichannel cognitive MAC protocol called CogMOR-MAC for multiple UAV networks, which is based on the multichannel opportunistic reservation mechanism was presented in [48]. The protocol primarily focuses on solving rendezvous problems. Furthermore, it improves negotiation efficiency using only one radio. A performance evaluation indicated that it can adapt to primary-user (PU) environments and allows reliable communication.

The usage of a hybrid mechanism to enjoy the benefits of both contention-based and contention-free is a very interesting research topic to handle MAC challenges. A hybrid MAC protocol of CSMA/CA and TDMA is presented in [21] to handle collision. It consists of a master UAV and multiple actor UAVs. It handles the collision by using a partnership-based mechanism between the two nearby UAVs. This mechanism helps the UAVs to transmit data packets with low delay and fewer collisions. On the other hand, the usage of machine learning techniques and artificial intelligence can increase scalability and reliability. The authors in [49] presented an adaptive demodulation-free random access (DFRA) scheme, in which an adaptive feature extraction algorithm is based on the current channel condition. DFRA also utilizes a support vector machine (SVM)-based technique to identify suitable MAC protocols for different purposes. An energy-efficient and location-aware MAC protocol is introduced in [50] for quality of service (QoS) enhancement in UAV networks. Collision is avoided between UAVs by using accurate positioning beacons, and congestion is avoided by harmonizing the transmissions.

## 4. Design Considerations for MAC Protocols of UIoT

The UIoT systems have their own unique features and challenges. For instance, the latency of data transmission between the IoT devices and UAV is usually limited because they communicate with each other through direct links rather than multi-hop communications. One of the biggest challenges faced by UIoT is that the MAC protocol should guarantee high network throughput and low energy consumption. Because most of the IoT devices and the UAV are battery powered, it is very important to make the system energy-efficient. At the MAC layer, energy wastage occurs due to collisions, idle listening, overhearing, and control packet overhead. To develop a new MAC protocol for UIoT, it is essential to analyze the parameters that significantly affect the performance of the MAC protocol. Hence, designing a novel MAC protocol for UIoT is a paramount topic for researchers. In this section, design issues related to the MAC protocol of UIoT are briefly discussed.

### 4.1. Throughput

Throughput is the measurement of data transmissions from source to destination at any given time. Achieving high efficiency and high throughput are major concerns in the development of a MAC protocol for a UIoT network. The shared spectrum for each device in a UIoT network is limited; therefore, the MAC protocol for UIoT should be capable of reducing collisions. Meanwhile, high throughput is necessary for accommodating millions of devices in the network. In a contention-based system, channel-access collisions may result in low throughput. Moreover, the hidden-node problem exacerbates the collision problem considerably. In a contention-free MAC protocol, empty slots and the control overhead affect the throughput of the overall system. Additionally, there is a tradeoff between the system throughput and energy efficiency [51], which must be acknowledged.

### 4.2. Energy Efficiency

Energy efficiency refers to the minimum energy usage to perform a task, thus eliminating energy wastage. The energy efficiency is an important design consideration for UIoT networks because UAVs, onboard sensor nodes, IoT devices, and ground sensor nodes are battery-operated and energy-constrained. Moreover, the power consumption due to environmental factors is not negligible. Two factors significantly affect the power consumption of battery-powered IoT devices and sensors: radio transmissions, and channel access. However, the amount of power consumed by the MAC layer can be reduced, e.g., by reducing the amount of idle listening, duty cycling [52], implementing sleep scheduling [53]; and assigning priority slots for low-power devices [54].

### 4.3. Low Latency

Low latency is very important when a huge amount of data is delivered with limited delay. In time- and mission-critical applications, it is very crucial to achieve low latency during data transmission [55]. It is because severe damage to the organization or personnel can occur if the delay is quite long. Thus, low latency is a highly important factor in designing MAC protocols for UIoT systems. 

### 4.4. Scalability

Scalability is a characteristic of an organization, system, model, or function that describes its capability to cope and perform well under an increased or expanding workload or scope. In a heterogeneous UIoT network, network scalability is critical. It is because millions of diverse devices and sensors are connected to a UIoT system. Therefore, the network should be able to extend itself when a new device of any type enters or leaves the network [56]. Moreover, the collaboration between UAVs and IoT devices or sensors can significantly improve system performance. Therefore, in a UIoT system, the use of multiple UAVs can improve network performance.

### 4.5. UAV Speed

The speed of any device is concerned with the mobility of the particular device. In a UIoT network, the speed of UAV has a significant impact on the channel-access mechanism. It is because when a UAV flies at a high speed, the duration of contact between the UAV and IoT devices is very limited [57]. Many IoT devices cannot access the channel in this short period; thus, the UAV speed must be optimized in such a way that all the devices get sufficient access time.

## 5. MAC Protocols for UIoT

**Figure 2:**
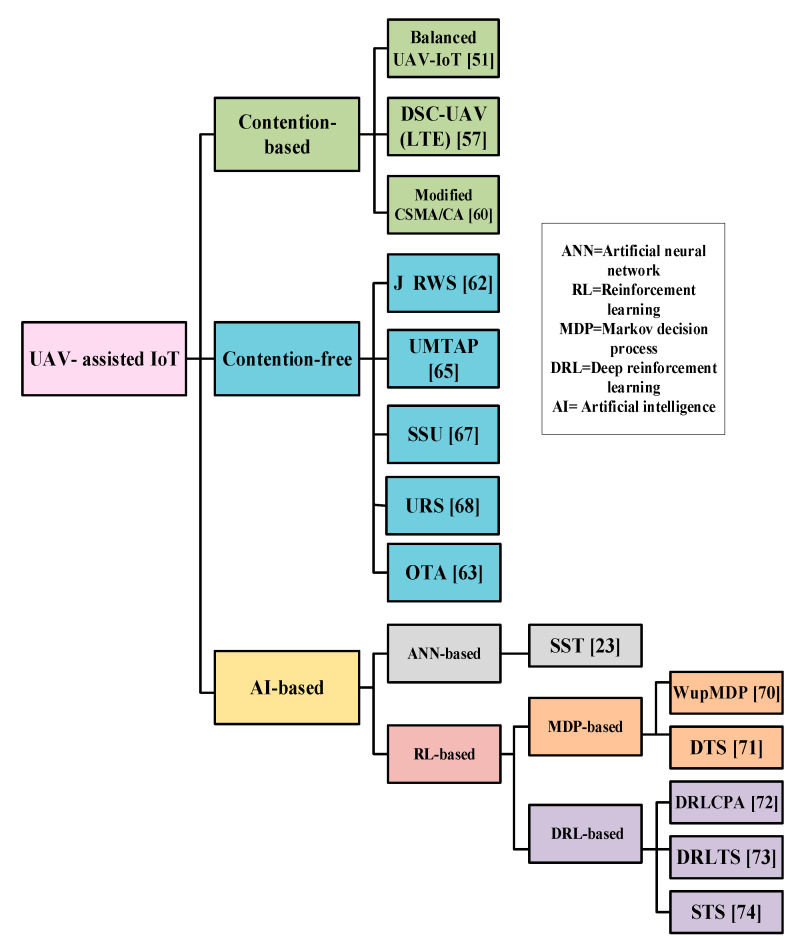
Taxonomy of the medium access control (MAC) protocols for UIoT.

In this section, the existing MAC protocols for UIoT are classified into three different categories as shown in Figure 2. They are classified according to their dominating characteristics. Most of the MAC protocols use a contention-based approach or contention-free approach. However, due to the rapid improvement of artificial intelligence-based MAC protocols are getting much attention. The MAC protocols reported thus far are categorized into three types: contention-free, contention-based, and artificial intelligence (AI)-based. In this section, the existing MAC protocols for UIoT are investigated with regard to their key features, operational characteristics, advantages, limitations, application domains, and potential improvements. This section is organized as follows: First, we discuss the contention-based MAC protocols for UIoT in Section 5.1. Then, we present the contention-free MAC protocols for UIoT in Section 5.2. Finally, we describe all the AI-based MAC protocols for UIoT in Section 5.3.

### 5.1. Contention-Based Protocols for UIoT

Contention-based protocols are considered as the simplest protocols with regard to setup and implementation. A contention-based protocol allows several users to use the same radio channel without preordination [58]. In a contention-based protocol, several nodes contend to transmit data to the same channel. The lack of scalability is regarded as a key disadvantage of this protocol because, when the number of nodes increases, the number of collisions increases owing to concurrent transmissions from different nodes. In this section, the existing contention-based UIoT MAC protocols are briefly discussed.

#### 5.1.1. Balanced UAV-IoT

**Figure 3:**
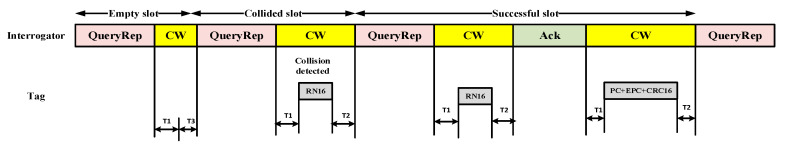
Link timing for the anti-collision mechanism in framed slotted ALOHA.

Xiaohui et al. [51] proposed a UIoT-based data-collection system for hostile, inaccessible areas. The main objective of their study was to maximize the energy efficiency of the whole system. This protocol utilizes a framed slotted ALOHA-based approach for data collection. In every frame, the radio frequency identification (RFID) reader of UAV broadcasts a “Query” command that includes the frame length at the start of each round of data transmission. After receiving the command, the nearby tag selects a slot in the frame independently and transfers it randomly. The position of the slot is used as a transmission counter. When the count reaches zero, data are sent immediately. Meanwhile, it can be observed in Figure 3 that the reader uses the “QueryRep” command to begin a slot. Each tag decrements its count by 1 when the command is heard. This RN16 packet is generated when the count reaches zero. A collision occurs when multiple tags send RN16 packets simultaneously and the slot is wasted. The slot is regarded as empty if no RN16 packets are sent. The reader sends an “ack” packet to the tag if it receives the RN16 packet, which indicates the successful reservation of the slot. Finally, the tag transmits its stored data to the UAV, which transfers the data to the end-users, including the PC, evolved packet core (EPC), data storage, and CRC-16.

The system efficiency ($\eta_{s}$) of the protocol can be defined as follows:

(1) $$\eta_{s}=\frac{a_{s}T_{s}}{a_{0}T_{0}+a_{c}T_{c}+a_{s}T_{s}},$$

where $a_{0}$ represents the expected number of empty slots in a frame, $a_{s}$ represents the expected number of successful slots in a frame, $a_{c}$ represents the expected number of collided slots in a frame, $T_{0}$ represents the duration of an empty slot, $T_{c}$ represents the duration of a collided slot, and $T_{s}$ represents the duration of a successful slot.

- Advantages: The Balanced UAV-IoT system has a high throughput in the system-efficiency mode, but its energy consumption is significantly lower in the energy-efficiency mode. Moreover, a cross-layer architecture is applied that helps to optimize different system parameters.
- Limitations: The system performance is significantly affected by the empty slots, collided slots [59], and overhearing.
- Application domains: The sensor nodes follow a line model, making the system suitable for railway lines, power lines, country borders, and human-inaccessible or hostile areas.
- Future improvements: The system efficiency can be enhanced by applying suitable path planning and a suitable UAV flight time.

#### 5.1.2. Dynamic Speed Control and Data Collection Framework of UAV (DSC-UAV)

**Figure 4:**
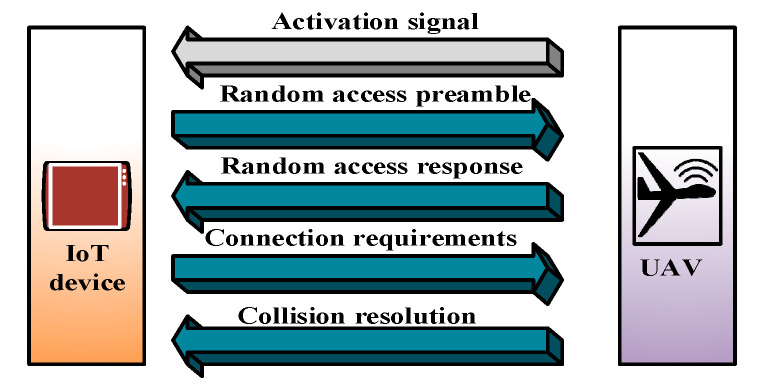
Random access procedure (RAP) with four handshaking steps.

Qi Pan et al. [57] proposed a data-collection framework for UIoT. Their main objective was to maximize the data-collection efficiency by adaptively controlling the speed of the UAV. The time is divided into frames of fixed length, and each frame consists of 10 sub-frames. Each sub-frame is further divided into two timeslots. The random-access procedure (RAP) can only be started in fixed timeslots. It is clear from Figure 4 that the connection between the devices and the UAV is established after four handshaking steps for data transmission. At the initial step of connection establishment, a physical random access channel (PRACH) is used for connection establishment. The IoT devices randomly choose available preamble sequences and transmit them to the UAV as an access request. However, when multiple devices select the same preamble sequence, the UAV cannot differentiate between them, and collisions occur. The UAV sends a random access response (RAR) to the IoT device after receiving the preamble.

However, if the IoT device within a time limit does not receive the RAR message, the connection fails and retransmission is necessary. After receiving the RAR from the UAV, the IoT device sends a Layer 2/3 (L2/L3) message. Collided devices face conflicts when transmitting L2/L3 messages. After receiving the message, the UAV sends an acknowledgment to the devices, and the connection is established. The devices that do not receive the acknowledgment enter the “sleep” mode and perform “backoff” to reinitiate the RAP until retransmission. After the connection is established, data transmission is completed. The UAV flies across the area with speed v, the number of newly arriving access requests (ΔM) can be derived as

(2) $$\Delta M\;=\;v\times T_{RA\_REP}\times \lambda \times 2k,$$

where $T_{RA\_REP}$ represents the interval between two consecutive random-access slots, λ per square meter refers to the drone’s trajectory, and the width of the area can be regarded as 2k. The arrival and collision rates are low for the connection establishment when the UAV flies at a low speed. When a UAV flies at a high speed, the number of newly arriving access attempts is large, causing the network to suffer from heavy congestion and overhead. Consequently, a collision occurs, and sensors cannot transmit their data efficiently. Hence, a speed-control algorithm for UAVs with a PRACH was developed.

- Advantages: DSC-UAV performs well with regard to the probability of successful access, access delay, and data-collection efficiency. Moreover, the optimal velocity of the UAV is measured for different densities of IoT devices.
- Limitations: DSC-UAV requires a four-step handshaking technique to establish a connection between the UAV and the IoT devices. However, owing to the dynamic and mobile nature of the UAV, a long connection process is undesirable. Many IoT devices can miss the opportunity for data transmission because of the long connection process.
- Application domains: This protocol may be suitable for a smart city where sensor nodes on roadsides (roads with sensors and smart vehicles) are higher than nearby smart buildings.
- Future improvements: The multi-UAV mechanism should be investigated for different densities of IoT devices. The multi-UAV systems can be compared with the DSC-UAV in terms of the system performance.

#### 5.1.3. Modified CSMA/CA

**Figure 5:**
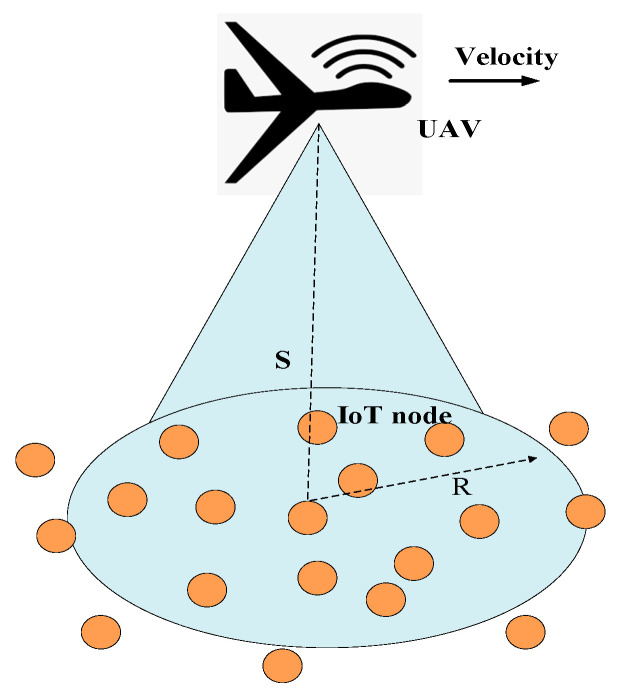
UAV flies in a straight trajectory to collect data from multiple IoT devices on the ground.

The authors in [60] investigated a CSMA/CA-based MAC protocol for a UIoT network. As shown in Figure 5, the UAV flies in a straight trajectory. The UAV is equipped with a directional antenna. Therefore, it projects a circle-shaped communication range while flying. The devices at different locations can access the UAV for different durations. 

The communication duration time between the IoT device and the UAV can be calculated as follows:

(3) $$T_{i}=\frac{2R\cos\theta_{i}}{v},$$

where R is the radius of the UAV coverage, v is the velocity of the UAV, and $\theta_{i}\in \left(0,\frac{\pi }{2}\right)$ is the location of the device and can be calculated as $\theta_{i}=\arcsin(\frac{y_{i}}{R})$. The channel-access procedure follows a Markov process. The devices contain different states, and a larger number of times that a device can traverse the whole Markov chain corresponds to a higher probability of successful transmission. The devices are divided into different band-shaped clusters that are parallel to the flight trajectory of the UAV. The contention-window (CW) size for each device in each cluster adaptively adjusted according to the contact duration. For maintaining fairness among the IoT devices, if the contact duration of a device is short, the size of the CW is small so that the device can access the channel before other devices having a longer contact duration. Additionally, the initial CW size and the retry limit adjusted according to the contact durations of each cluster. The initial CW size for each device in a cluster is calculated as follows:

(4) $$CW_{\min}^{i}=[(1-\frac{t_{i}}{T}\times CW_{\min}^{\max}],$$

where $t_{i}=i\times \Delta$ represents the contact duration for a cluster, and T represents the longest contact duration for a cluster, $CW_{\min}^{\max}$ represents the *CW* size, the range of which is between a maximum CW and a minimun *CW* values.

- Advantages: The Modified CSMA/CA protocol outperforms the original CSMA/CA with regard to throughput for different densities of IoT devices owing to the use of transmission priorities.
- Limitations: The CW size must be calculated and updated frequently, leading to energy and time consumption for the system.
- Application domains: Wide, remote areas with randomly distributed IoT devices are suitable candidates for this protocol.
- Future improvements: Hidden and exposed nodes must be addressed to reduce collisions and energy wastage.

### 5.2. Contention-Free MAC Protocols for UIoT

In a contention-free MAC protocol, collisions are avoided by ensuring that each node can use its allocated resources exclusively [61]. The main advantage of using a contention-free protocol is that it can utilize the channel more effectively than a contention-based protocol at high load. However, when the network size changes, it is difficult for the protocol to adapt. Regarding the UIoT network, the disadvantages of the protocols outweigh the advantages in terms of flexibility and scalability. However, contention-free protocols that can dynamically adapt to different network sizes are suitable for UIoT networks. In this section, the existing studies on the MAC protocols for UIoT are discussed.

#### 5.2.1. Joint Resources and Workflow Scheduling (JRWS)

**Figure 6:**
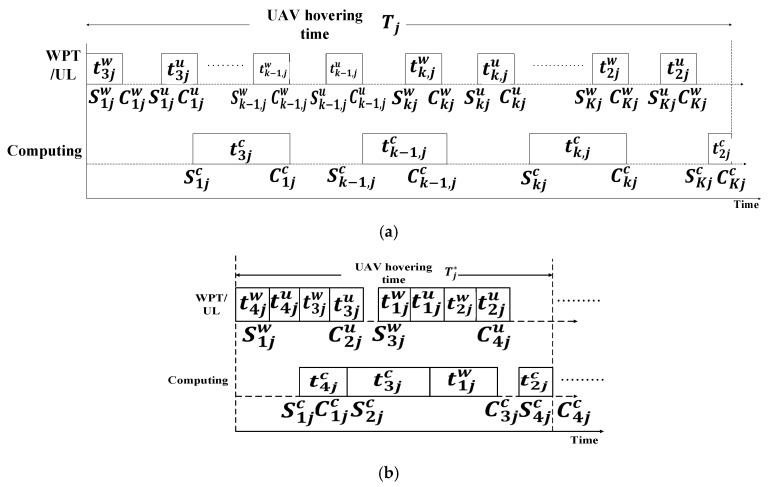
Time-division multiple access (TDMA)-based workflow model in Joint Resources and Workflow Scheduling (JRWS). (**a**) Random service flows after scheduling; (**b**) random service flows after optimization.

A UAV-enabled MEC system wherein the UAV is equipped with WPT technology was investigated in [62]. The main objective of that study was to minimize the total energy consumption of the UAV by jointly optimizing the IoT device association, resource allocation, UAV hovering time, WPT duration, uplink data rate, and service sequence of the IoT devices. The UAV can perform multi-user tasks such as WPT, communication, and computation. In one flying mission, a UAV must complete three different tasks: WPT from the UAV to the IoT devices, data uploading from the IoT devices to the UAV, and data processing. These three stages are denoted as $t_{ij}^{w},t_{ij}^{u},$ and $t_{ij}^{c}$, respectively. The workflow is executed via TDMA. After reaching the destination, the UAV sends a signal to an IoT device to switch its working mode to the energy harvesting (EH) model for harvesting radiofrequency energy. Subsequently, the UAV transfers power to the IoT devices via WPT. Finally, the IoT devices upload their data to the UAV. The TDMA-based workflow model is shown in Figure 6. Here $T_{j}$ represents the j-th hovering time of the UAV, and $S_{j}$ represents the service sequence of the IoT device that selects the jth UAV hovering location to upload its data. From Figure 6a, it is visible that when the optimization of workflow is absent, the UAV provides services to the IoT devices in a random fashion. Moreover, there remains some idle time between the first data uploading time and the first task computing time. It is poorly ordered compared with the optimized workflow shown in Figure 6b. In Figure 6b, it can be seen that after the optimization of workflows, there seems to be no idle time between data uploading and computing which significantly improves the system efficiency. The three tasks—power transfer, data transmission, and task execution are performed sequentially. None of these tasks can be interrupted. Therefore, the workflow can be defined as follows:

(5) $$s_{kj}+t_{kj}=c_{kj},k\in K_{j},\forall_{j}\in M,$$

where $s_{kj}=\left[s_{kj}^{w},s_{kj}^{u},s_{kj}^{c}\right]$ represents the starting-time vector of each operation in the kth service flow, $t_{kj}=\left[t_{kj}^{w},t_{kj}^{c},t_{kj}^{u}\right]$ represents the duration vector of the three service stages in the k-th workflow, and $c_{kj}=c_{kj}^{w},c_{kj}^{u},c_{kj}^{c}$ represents the completion-time vector of the operation corresponding to $s_{kj}$, where $k\in K_{j}$. As illustrated in Figure 6, the data uploading stage is after the wireless powering stage, and the task computing stage is after the data uploading stage. Therefore,

(6) $$\{\begin{array}{c} s_{kj}^{w}\geq 0\\ s_{kj}^{u}\geq c_{kj}^{w},k\in K_{j},\forall j\in M\\ s_{kj}^{c}\geq c_{kj}^{u} \end{array},$$

Moreover, there is at most one IoT device (IoTD) being served at one time in each workflow, which means that the k-th stage is after the (k−1)-th stage. Thus,

(7) $$\{\begin{array}{c} s_{kj}^{w}\geq c_{k-1,j}^{w}\\ s_{kj}^{u}\geq c_{k-1,j}^{u},k\in K_{j},\forall j\in M\\ s_{kj}^{c}\geq c_{k-1,j}^{c} \end{array}$$

The IoT device association, resource allocation, UAV hovering time, and service duration are optimized mathematically, and the optimal algorithm is obtained. Subsequently, all the algorithms are merged to develop the final iterative joint resources and workflow-scheduling scheme.

- Advantages: The proposed workflow model provides a multitasking mechanism during the UAV flight. This helps to reduce the UAV hovering time and energy consumption.
- Limitations: Large synchronization overhead is suspected for the communication between the UAV and the IoT devices due to the use of the TDMA structure for multi-workflow modeling.
- Application domains: This algorithm can be used in industrial IoT applications where a multiple-workflow system is necessary to reduce the time consumption. Furthermore, it may be suitable for emergency cases where multiple-workflow algorithms can reduce the time and energy consumption.
- Future improvements: The multichannel mechanism can be applied for improving the channel utilization.

#### 5.2.2. Optimal Time Allocation (OTA)

**Figure 7:**
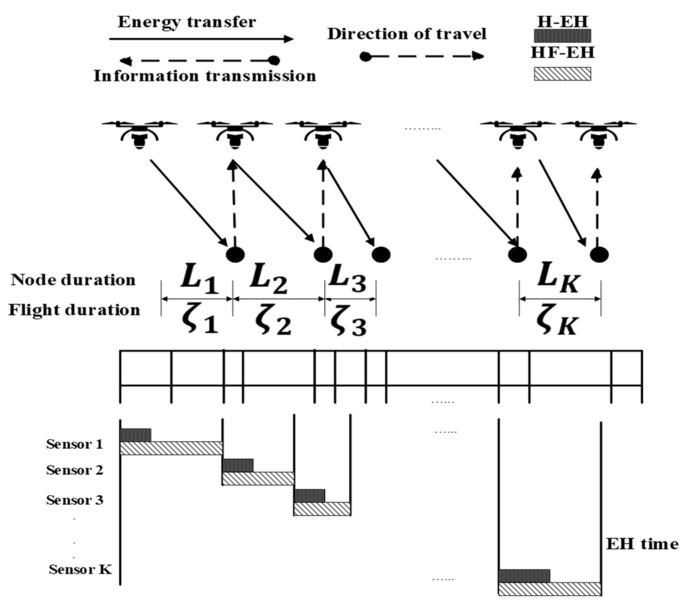
Line model and frame structure for energy harvesting (EH) and information transmission.

Han-Ting Ye et al. [63] presented an OTA scheme for UAVs based on a full-duplex wireless-powered IoT network. Their main objective was to maximize the network throughput. A dynamic TDMA frame structure is employed, where data transmission can only occur during each sensor’s allocated timeslot. During the data collection, the UAV receives the uplink information from the current sensor through one of the antennas. The other antenna is used for transmitting radio signals (with a constant transmit power) to the neighboring sensor nodes. The line model and frame structure for EH and information transmission are presented in Figure 7. Figure 7 shows that the sensor nodes only get the opportunity to transmit their data to the UAV when UAV is within their communication range. LoS connection between sensors and UAV is also shown. It is also clear from the figure that the sensor nodes can only transmit on their designated time slot. The uplink channel gain from sensor i to the UAV is given as $h_{i}=\frac{k_{0}}{A^{2}}$, and the downlink channel gain can be expressed as $g_{i}=\frac{k_{0}}{A^{2}+L_{i}^{2}}$, where $k_{0}$ represents the signal power gain at a distance of 1 m from the sensor, A represents the altitude of UAV, and L represents the distance between two neighboring sensors. The total harvested energy for sensor i can be expressed as follows:

(8) $$E_{i}=\eta_{i}g_{i}P_{t}\tau_{i-1}+\int_{0}^{\varsigma_{i}}\frac{\eta_{i}k_{0}P_{t}}{A^{2}+{\left(L_{i}-v_{i}t_{g}\right)}^{2}}dt_{g},$$

where $\eta_{i}\in (0,1)$ is a constant that represents the EH efficiency for sensor i, and $v_{i}=\frac{L_{i}}{\varsigma_{i}}$ represents the constant speed of the UAV flying from sensor i−1 to sensor i, $P_{t}$ represents the power transmitted by the UAV antenna, $\tau_{i}$ represents the hovering time of the UAV, $\varsigma_{i}$ represents the flight time, and d represents the distance between UAV and sensor devices. The total throughput of the OTA scheme is determined by solving a convex optimization problem. This problem is solved numerically using the Karush–Kuhn–Tucker (KKT) conditions. Finally, the algorithm for OTA is derived.

- Advantages: OTA can achieve the maximum throughput within an optimized allocated time. Additionally, the UAV wirelessly transfers energy to sensors while hovering and flying.
- Limitations: IoT networks tend to be dense [64]. However, the system was not simulated in a dense IoT network. The system performance highly depends on the density of IoT devices in the network.
- Application domains: This model is suitable for IoT systems that are located in remote and rural areas and deployed in a line. The UAV can facilitate the sensors by acting as a charging source.
- Future improvements: The UAV altitude, packet size, and number of nodes should be optimized for improving timeslot access.

#### 5.2.3. UAV-Based Multiple Tags Anti-Collision Protocol (UMTAP)

**Figure 8:**
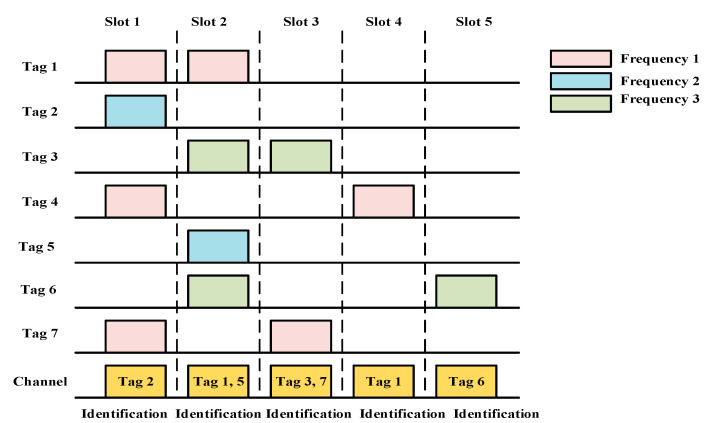
Multi-frequency tags collision.

The authors of [65] proposed a UAV-based anti-collision algorithm for dense RFID-based IoT networks. Their main objective was to avoid collisions among the multiple RFID tags. The time is divided into numerous timeslots. However, a collision occurs when tags with the same frequency attempt to access the channel in the same timeslot. The expected value of the collision in each frame is given as:

(9) $$R_{c}=\frac{C_{s}}{L}=1-{\left(1-\frac{1}{L}\right)}^{N}(1+\frac{N}{L-1}),$$

where $C_{s}$ represents the collided slot, L represents the frame length, and N represents the number of tags. The UMTAP follows two steps for avoiding collisions between the tags: optimizing the system efficiency and achieving multi-frequency tag communication. The system efficiency depends on the optimal frame length of the system. A frame length closer to the number of tags corresponds to higher system efficiency. However, when the number of tags is >256, the system efficiency cannot be increased by increasing the frame length. To solve this problem, identification of tags according to the successful slots, empty slots, and collisions is necessary. Finally, the system efficiency can be calculated using the following equation:

(10) $$E_{systemUMTAP}=\frac{S_{Snew}}{L},$$

where $S_{new}$ represents the number of successful timeslots and L represents the frame length. The main focus of the work is to use multi-frequency tags to avoid collisions in the same time slot. As shown in Figure 8, the multiple tags of different frequencies can be identified in the same time slot. However, collisions can only occur when the same frequency tags transmission occurs in the same time slot. For example, in slot 2, tags 1 and 5 are identified as they use different frequency levels. However, tags 3 and tag 6 cannot be identified because they use the same frequency level.

- Advantages: The anti-collision mechanism of the UMTAP yields a high system efficiency. Additionally, the tag identification time is shorter than that of similar protocols.
- Limitations: Channel and energy wastage occurs owing to the empty slots [66]. The numbers of successful slots, empty slots, and collision slots must be calculated after every one-slot duration, leading to significant energy and time consumption. Moreover, the speed and mobility of the UAV are not considered.
- Application domains: This protocol is useful for dense RFID-based environments in a 3D communication scenario.
- Future improvements: The multi-UAV architecture should be applied to increase the coverage area, and a mechanism of acquiring data from the IoT nodes should be developed.

#### 5.2.4. Spectrum Sensing Using UAV (SSU)

**Figure 9:**
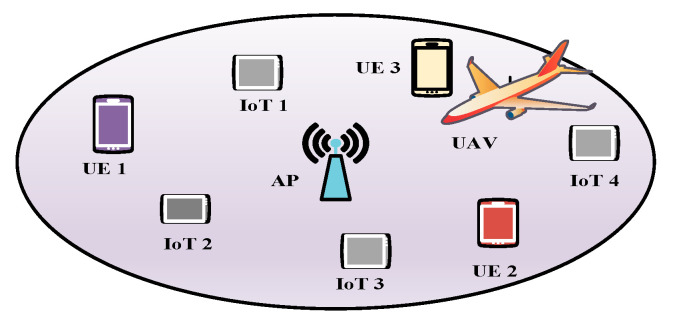
Data dissemination to IoT devices using a cognitive UAV.

The authors in [67] proposed a data-dissemination mechanism for a UIoT network using a cognitive UAV. Their main objective was to maximize the number of bits received by the IoT devices from the UAV. Figure 9 shows that numerous user equipment (UE) and IoT devices are deployed in the target area. The UAV serves all the IoT devices in frame T, and each frame is divided into numerous slots. In each frame, the UAV attempts to sense a channel to find an idle channel. Before the end of the frame, the UAV selects an idle channel for data transmission. If an idle channel is found, the UAV begins to transmit the data immediately in $n_{i}$ consecutive slots among the N slots. 

If no idle channel is found, the UAV uses the Industrial, Scientific, and Medical (ISM) band to transmit the data for supporting delay-sensitive applications. After transmitting the data to the IoT devices, the UAV uses the remaining time for channel sensing and moving. Each IoT device must be synchronized with the UAV and receive control information over a Common Control Channel (CCC). After selecting an idle channel, the UAV broadcasts information about the selected channel to the nearby IoT devices over a CCC channel. After receiving the information, the IoT devices turn on their radio to receive data from the UAV over the selected channel. Then, the IoT devices confirm the availability or unavailability of the channel to the UAV. If an IoT device finds that the channel is busy, it sends a negative acknowledgment (NAK) signal to the UAV over the CCC channel. Thus, the UAV understands that the channel is not available when at least one IoT device sends an NAK signal over the CCC. If the UAV finds that multiple channels are idle, it selects the channel with the longest elapsed time of the OFF period. The height of the UAV is H, the feasible sensing region is specified by the area between $D_{l}$ and $D_{m}$. Hence, the UAV can sense the spectrum when its horizontal location is between $D_{l}$ and $D_{m}$. Hence, it can be written as

(11) $$D_{l}\leq \left\|L(t)-L_{B}\right\|\leq D_{m},$$

where L(t) represents the location of the UAV at the beginning of the frame t, and $L_{B}$ is the location of the base station. Accordingly, $D_{l}$ and $D_{m}$ can be derived respectively by

(12) $$D_{l}=\frac{H_{B}-H}{\left|\tan(\theta_{Tilt}+\frac{\theta_{Beam}}{2})\right|}$$

and

(13) $$D_{m}=\frac{H_{B}-H}{\left|\tan(\theta_{Tilt}-\frac{\theta_{Beam}}{2})\right|},$$

where $H_{B}$ is the height of the base station, $\theta_{\frac{Beam}{2}\;+\;}\theta_{Tilt}<90$, and $\theta_{Tilt}\neq \frac{\theta_{Beam}}{2}$.

- Advantages: The proposed mechanism performs well in terms of system throughput and avoids collisions due to the use of the cognitive UAV to detect idle channels.
- Limitations: The UAV continuously senses the channel while moving, leading to significant energy consumption.
- Application domains: Remote areas with multiple mobile users are promising candidates for this protocol.
- Future improvements: An optimized and efficient trajectory should be designed for the UAV to locate the IoT devices efficiently without significant energy wastage.

#### 5.2.5. UAV-Based Relaying and Scheduling (URS)

**Figure 10:**
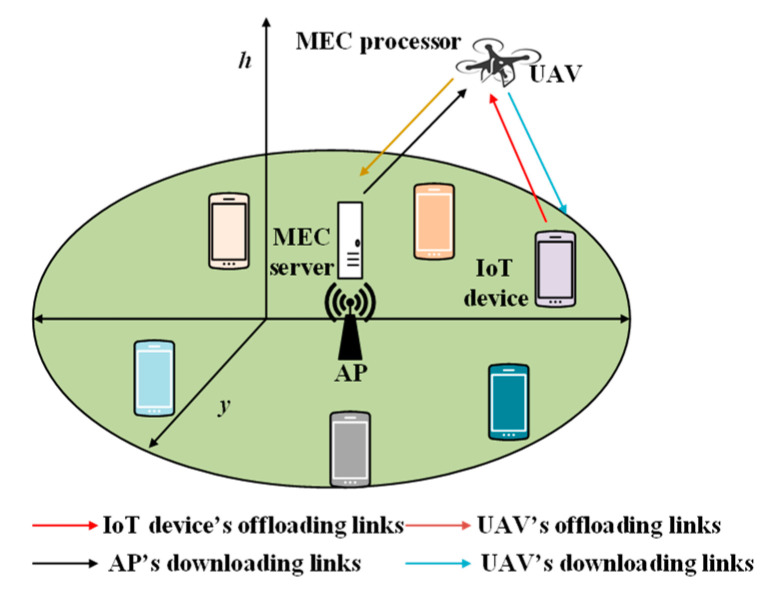
UAV functions as an MEC server to help the ground IoT devices perform their computational tasks or as a relay to forward the offloaded tasks to the AP.

The authors in [68] proposed a UAV-based MEC system where the UAV functions as both a computing server and a relay for ground IoT devices. Their main objective was to minimize the total energy consumption of the UAV and ground IoT devices. Figure 10 shows the scenario of the proposed system: a cellular connected UAV, an access point (AP), and ground IoT devices. 

In this system, each ground IoT device can perform its computational task in three ways: local computation, task offloaded to UAV, and task offloaded to the AP. In the first case, the IoT device performs the tasks locally, by itself. The number of computation bits and the energy consumption of the IoT devices during timeslot *n* for local computation are given as follows:

(14) $$L_{k}^{local}[n]=\frac{\tau f_{k}[n]}{C_{k}},k\in K,n\in N$$

and

(15) $$E_{k}^{local}[n]=\tau k_{k}f_{k}^{3}[n],k\in K,n\in N,$$

respectively, where $f_{k}\left[n\right]$ represents the central processing unit frequency during timeslot n, $C_{k}$ represents the required amount of computational resources, N represents the location of the IoT devices, τ represents the duration of the timeslot, and *N* represents the number of timeslots. In the second mechanism, the IoT device can partially offload its task to a nearby UAV. During the process of task offloading from the IoT device to the UAV, TDMA is used to prevent collisions among the IoT devices. Each slot of TDMA is divided into K equal time divisions. In each slot, the IoT devices upload k bits to the UAV. In the third mechanism, latency-critical tasks are offloaded to the AP for faster computation. After the computation is complete, the AP returns the computation results to the UAV in a TDMA-based manner, using a different bandwidth. The UAV transmits the computation results to the ground IoT devices.

- Advantages: By performing simulations, the UAV trajectory has been optimized for different scenarios. The optimization algorithm can yield stable performance in a dynamic environment.
- Limitations: Latency-critical tasks are not prioritized over normal tasks in the computation process. Hence, the completion of latency-critical tasks may be delayed if the number of normal tasks is large.
- Application domains: Remotely deployed IoT devices with blocked surroundings are suitable for this protocol.
- Future improvements: The multi-UAV scenario should be adopted for better coverage and interference avoidance between UAVs.

### 5.3. AI-Based MAC for UIoT

AI is being increasingly used in communication networks [69]. It allows the system to make more precise by interacting with the environment. On the other hand, reinforcement learning (RL) is one of the most widely studied topics in the field of AI and attracts increasing research attention. RL is a machine-learning technique that allows an agent to learn in an interactive environment in a trial-and-error manner, using feedback from its own actions and experiences. On the other hand, in deep reinforcement learning (DRL), the principles of deep learning and reinforcement learning are used to create an efficient algorithm. This method has been used to solve a wide range of complex decision-making tasks that could not be solved previously by machines. Finally, the Markov decision process (MDP) is a memoryless random process. It consists of four components of states, actions, transition probabilities, and reward. It is widely used by researchers because it is a discrete-time state transition system. In this section, all kinds of AI-based MAC protocols: of ANN, MDP, and DRL-based MAC protocols are discussed in detail.

#### 5.3.1. Sustainable and Secure Trajectory (SST)-Based MAC for UIoT 

**Figure 11:**
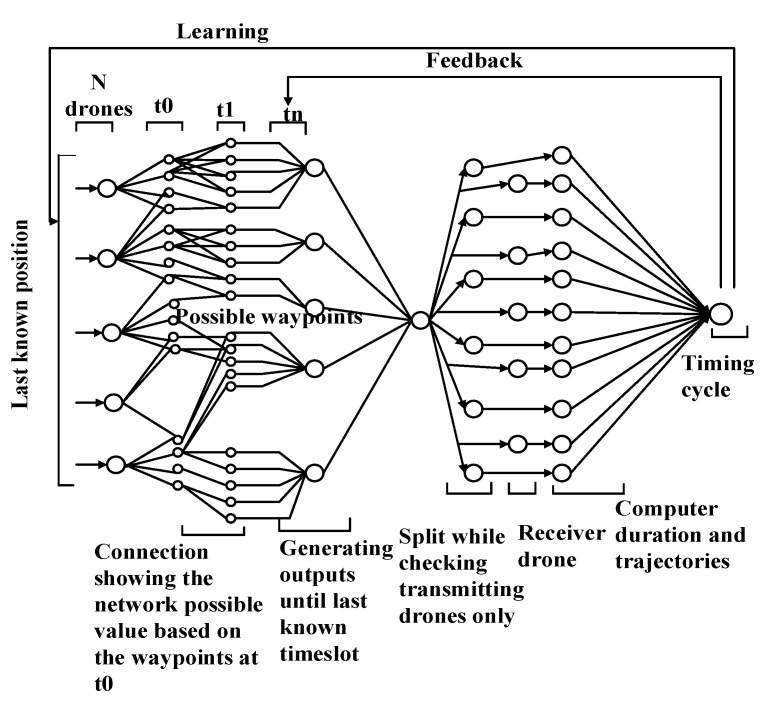
Illustration of the deep neural network model for generating secure and non-overlapping trajectories for UAVs in control with the timing procedures of the MAC protocol.

The SST-based MAC, which was proposed in [23], is a deep neural network-based security framework. The main objective of the study is to secure the trajectory of UAV by maintaining link stability. The framework is embedded with an efficient MAC protocol that is controlled by the Macaulay duration. The deep neural network-based framework of MAC can generate trajectories and estimate paths. To prevent collisions, the waypoints are calculated dynamically. First, the network is used for building an efficient MAC scheme, which increases the channel accessibility and provides timing control for relaying. After the MAC is operational, a neural network is employed to verify every connection in the network. Finally, the network generates highly reliable and secure coordinates for the military IoD. The system model uses three types of communication links: UAV-to-UAV, UAV-to-device, and virtual. Using these links, a secure trajectory is obtained. Based on the Macaulay-duration conditions, the timing principle for the MAC protocol is determined. According to the timing principle of each UAV, the values of the past, present, and upcoming states can be obtained. Furthermore, the timing principle can be used to select a UAV that will start transmitting data at a particular time, and mapping is performed to clearly identify the difference between the observed and expected values. The proposed model utilizes a supervised deep neural network-based framework that supports the pre-identification of faulty coordinates, which is based on expected and legitimate waypoints. It is clear from Figure 11 that the deep neural network-based MAC protocol comprises three steps: matching, feedback, and learning. In the first step, matching is performed between the MAC output and the deep neural network. This supports the accumulation of results for evaluating the progress of the UAVs and their communication capabilities. At the system level, a matching operation is performed, which generates a constant Θ for every variable. The system verifies whether it matches or violates the criteria. In the feedback phase, it is determined whether the model is in a state to continue. Increasing the number of iterations contributes to the generation of safe waypoints. Moreover, error ratings pertaining to the model performance are generated in the feedback phase. In the learning phase, learning is conducted via supervisory control. A probabilistic model is employed to identify the values to be used as the input for calculating each metric. The learning rate $L_{e,<p_{1}>}^{\left(t\right)}$, can be represented as follows:

(16) $$L_{e,<p_{1}>}^{\left(t\right)}=\frac{1}{N^{\prime }}\left(\beta_{e,<p_{2}>}^{\left(t\right)}\times \sum_{i=1}^{t}\left(k_{i}\times \frac{\beta_{e,p_{2}}^{\left(i-1\right)}}{\beta_{e,p_{2}}^{\left(i\right)}}\right)\right),$$

where $\beta_{e,<p_{2}>}^{\left(t\right)}$ represents the error in the metrics of link duration, resource consumption, and learning rate, and $k_{i}$ represents the learning constant, which is the controlling factor for the entire model. The ratio $\frac{\beta_{e,p}^{\left(t\right)}{}_{{}_{2}}}{\beta_{e,p_{2}}^{\left(i-1\right)}}$ depends on the dominance of the factor under evolution. $N^{\prime }$ represents the number of active UAVs. $p_{1}$ represents the present value of the learning rate, and $p_{2}$ represents the present value of the error in the metrics of link duration, resource consumption, and learning rate. If the denominator is weak, the ratio is preserved and the model is sustainable.

- Advantages: The protocol provides high performance, along with the optimal division of resources and link durations.
- Limitations: Owing to the complex system model, real-life implementation is difficult. Moreover, the model does not consider the energy consumption.
- Application domains: This protocol may be suitable for military and governmental applications, which requires secure path trajectories and sustainable links, because the system is secure from third-party attacks.
- Future improvements: The interference affecting the UAV-UAV communication should be considered by introducing a synchronization process between the UAVs.

#### 5.3.2. Wake Up-Based on MDP (WupMDP) for UIoT

**Figure 12:**
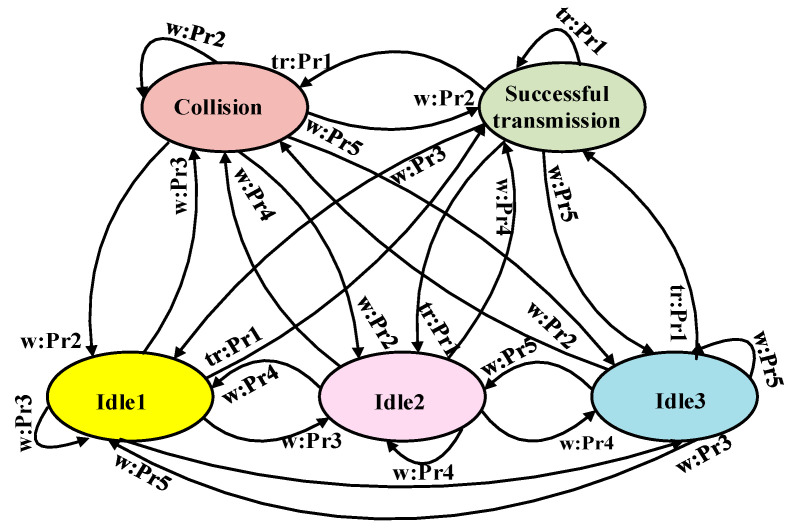
Collision handling on the wake-up plane using a Markov decision process (MDP).

Djiroun et al. [70] presented an MDP-based collision-avoidance model. Their main objective was to derive a decision policy to determine whether a UAV should transmit a wake-up signal to its ground devices within a specified range for data transfer or postpone the message transmission to the next slot. When multiple UAVs or multiple sensor nodes attempt to transmit a wake-up message to a single node, a collision may occur between the wake-up messages. Hence, an infinite-horizon time MDP was applied. To reduce the probability of collisions, two radio channels are used: a main radio and a wake-up radio. The wake-up radio is responsible for waking the main radio when it receives a wake-up message. The receiver’s wake-up radio uses the wake-up message to power itself on and activates the microcontroller that triggers the main radio. Figure 12 illustrates the MDP for collision handling on the wake-up plane. The authors considered a network composed of n devices, including UAVs and ground sensor nodes. It can be seen from Figure 12 that in a successful transmission phase, the decision-maker decides whether a wake-up message should be transmitted. If the decision-maker and other nodes attempt to send wake-up messages to the same destination, a collision occurs. Idle1, Idle2, and Idle3 are waiting states. If only one device among the (n−1) remaining devices decides to transmit and the others decide to wait, the agent enters the Idle1 state. The Idle2 state occurs when more than one of the remaining devices decide to transmit and a collision occurs. The Idle3 state occurs when both the decision-maker and the remaining devices decide to wait. The authors defined A=(tr,w) as a vector that represents the possible actions available for the decision maker in each state. The action tr indicates that the agent decides to transmit a wake-up message, and w represents the decision to wait. The probability of the packet transmission rate is denoted as P, which follows a Poisson distribution. $P_{r}\;\left(s^{\prime }|s,a\right)$ indicates the transition rate between states s and $s^{\prime }$ in S, considering a given action a, which represents the channel situation of successful transmission, collision, or idle states when n devices perform their actions. Therefore, the transmission probability $P_{r}$ can be denoted as:

(17) $$P_{r}\left(s^{\prime }|s,a\right)=\{\begin{array}{cc} {\left(1-p\right)}^{n-1} & s^{\prime }=succesfultransmission,s\in S\;\&\;a=tr\\ 1-{\left(1-p\right)}^{n-1} & s^{\prime }=collision,s\in S\;\&\;a=tr\\ (n-1)p{\left(1-p\right)}^{n-2} & s^{\prime }=idle1,s\in S\;\&\;a=w\\ 1-\left(\left(n-1\right)p{\left(1-p\right)}^{n-2}+{\left(1-p\right)}^{n-1}\right) & s^{\prime }=idle2,s\in S\;\&\;a=w\\ {\left(1-p\right)}^{n-1} & s^{\prime }=idle3,s\in S\;\&\;a=w \end{array},$$

- Advantages: WupMDP MAC is effective for successful packet delivery. Moreover, an efficient collision-avoidance mechanism is applied in the device wake-up period, helping to prevent collisions during data transmission.
- Limitations: The localization of the IoT devices, which is crucial for the UIoT network, is omitted. Moreover, the UAV speed, height, and path planning are not considered.
- Application domains: This protocol may be appropriate for IoT monitoring applications where sensors must send data occasionally, e.g., soil monitoring, crop-field monitoring, and flood monitoring.
- Future improvements: The proposed model and algorithm should be extended to the multi-UAV application scenario, and the interference among the UAVs should be addressed.

#### 5.3.3. Delay-Aware IoT Task Scheduling (DTS) with Constrained MDP (CMDP) for UIoT 

**Figure 13:**
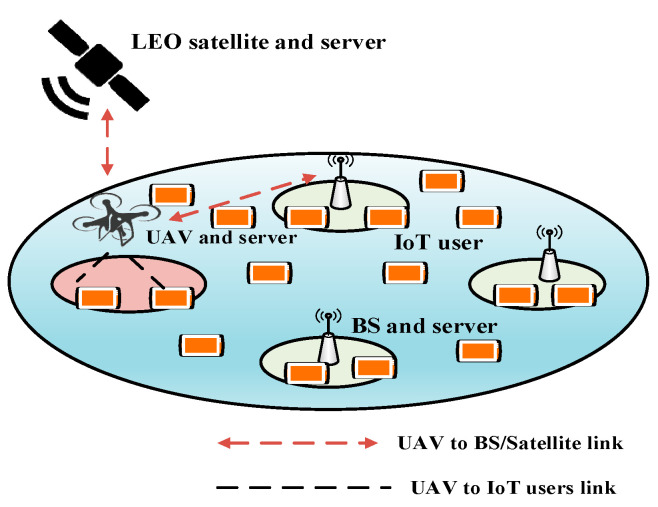
Network model of Delay-Aware IoT Task Scheduling (DTS).

The authors in [71] presented a SAGIN-based architecture for UAV-based IoT applications. Their main objective was to design an efficient task-scheduling algorithm for the dynamic environment of the UAV to minimize the long-term system delay. Figure 13 represents that the system is equipped with *N* number of BSs, one UAV, one LEO satellite, and a large number of IoT devices. The UAV is equipped with two interfaces: one is for communication with the satellite, and the other is for communication with the BSs and IoT devices. The timeslots have equal duration. Only one BS or satellite can be processed in each timeslot. The amounts of computational tasks offloaded from the IoT devices to the UAV differ among the timeslots.

After obtaining a task from an IoT device, the UAV decides whether to perform the computation locally or offload it to the satellite or a BS. If a large number of tasks are computed locally, newly arrived tasks are dropped. The link availability and task arrival are highly dynamic in a SAGIN. To make the decision of task offloading to a BS or satellite, an MDP is applied. The MDP problem is formulated as five parts: state space, decision space, state transition probabilities, reward function, and policy. The state of MDP depends on the UAV location and the current queue backlog. The decision space refers to the offloading decisions that are made based on the current state and the number of offloaded tasks. The state transition depends on the UAV trajectory and the queue backlog in the UAV buffer. The probability of the state transition is denoted by the following equation:

(18) $$p(s^{\prime }|s,a)=\{\begin{array}{c} f(H(q^{\prime }-v))\;case\;1\\ 1-F(H(q^{\prime }-v))\;case\;2\\ 0\;\;\;otherwise \end{array},$$

where s=(x,q),  s∈S describes the system state where x and q are the UAV location and the current queue backlog, respectively. A decision is made by the current state and the decision is denoted by a tuple a=(a,m),  a∈A, where a. is used to indicate offloading decision to which BS or satellite and m denotes the number of offloaded tasks. H denotes the number of time slots, f(.) and F(.) define the probability mass function and the cumulative distribution function of the new arrival task amount, respectively. Case 1 also indicates $x^{\prime }=x+H,\;v\leq q^{\prime }\leq \rho -1$, case 2 indicates $x^{\prime }=x+H,\;q^{\prime }=\rho$, ρ represents the buffer capacity, and v represents the number of tasks. The reward function is related to the computation delay of the offloaded tasks. The reward function r(s,a) on state s with action a is defined as follows:

(19) $$r(s,a)=-(d^{0}+d^{1}+d^{2}+d^{3}),$$

where $d^{0}$ means the computation delay of offloaded tasks, $d^{1}$ means the computation delay of tasks locally processed, $d^{2}$ indicates the waiting time of tasks not offloaded or not processed locally, and $d^{3}$ is the transmitting delay or offloaded tasks. According to the stationary deterministic policy, each state is assigned a fixed action, which is used whenever the system is that state. However, in a dynamic environment, is it important to find a stochastic policy in a CMDP. The problem of finding a stochastic policy is formulated as follows:

(20) $${}_{\pi }^{\max}\sum_{s\in S,a\in A}r(s,a)\pi (s,a),$$

where π represents the stochastic policy that includes all the steady-state probabilities π(s,a) for every accessible state-action pair, and r(s,a) represents the reward function for state s with action a. Later on, linear programming is used to find the stochastic policy in the CMDP.

- Advantages: The proposed scheme performs well with regard to the system delay for tasks with different arrival rates, owing to the use of the CMDP.
- Limitations: The interference between the UAV and the satellite is not considered.
- Application domains: Smart cities and smart oceans are good candidates for this scheme.
- Future improvements: Multi-UAV and multi-satellite scenarios should be considered to enhance the applicability.

#### 5.3.4. DRL-Based Channel and Power Allocation Framework (DRLCPA) 

**Figure 14:**
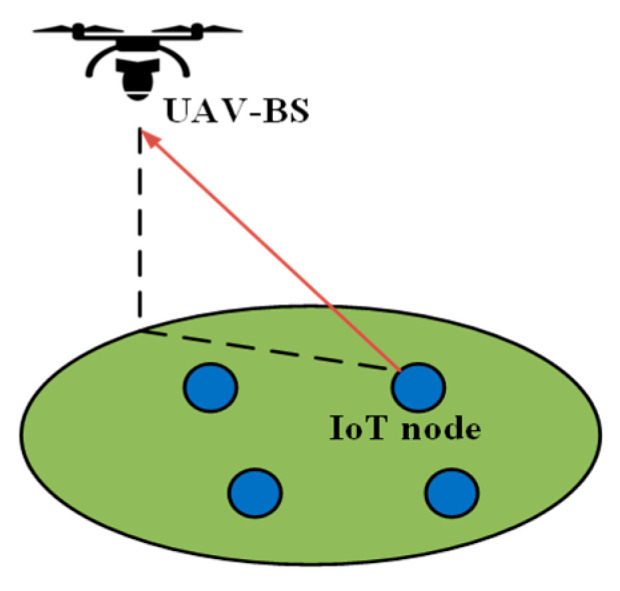
UAV is deployed to collect data from IoT nodes.

The authors in [72] proposed a DRL-based channel and power allocation mechanism for a UIoT network. Their main objective was to enhance the energy efficiency of the IoT devices by efficiently allocating both the channels and the power. As shown in Figure 14, a UAV is deployed to collect the data from the IoT devices. The time axis is divided into continuous equal-length time slots and each node is allocated with one channel for the uplink transmission in each time slot. It is assumed that the IoT devices produce data continuously. If multiple IoT devices are allocated on the same channel, the UAV adopts the TDMA mechanism for data transmission. The main focus of the work is to maximize the long-term energy efficiency from the current time slot to the future. This problem is formulated as an optimization problem. To solve this problem, an actor–critic DRL algorithm is adopted. The state space s(t) of the problem. To solve this problem, an actor–critic DRL algorithm is adopted. The state space s(t) of the proposed DRL framework is composed of the state of the UAV at timeslot t. In each timeslot, the UAV must allocate both channels and power to the IoT devices. Therefore, the action space contains M elements. The first group of M elements is $k_{i,t}\forall \;\in M$ and the second group of M elements is $P_{i,k_{i,t},t,}\;\forall \;\in M$. The action space a(t) denotes the action of the UAV in timeslot t. The action space of the UAV is defined by the following equation:

(21) $$a(t)=\left\{k_{0,t,}\ldots .k_{M-1,t}P_{0,k_{0,t},t}\ldots \ldots ,P_{M-1,k_{M-1,t,}t}\right\},$$

where $P_{i,k_{i,t}t}\in \left\{\frac{P_{\max}}{L},\frac{2P_{\max}}{L},\ldots ..,\frac{(L-1)P_{\max}}{L},P_{\max}\right\}$, and L>1 represents the number of power levels, $P_{max}$ means the maximum power. A reward is given to the UAV after each action. The reward is given as follows:

(22) $$r(t)=\eta_{t},$$

where $\eta_{t}$ represents the minimum energy efficiency among all the IoT nodes. The UAV functions as a DRL agent and utilizes two neural networks: the actor network and the critic network. The actor network helps the UAV to select an action, and the critic network is used to evaluate the reward based on the action. At the beginning of timeslot 1, the UAV establishes the actor and critic networks. Then, the UAV utilizes the established neural networks to make decisions regarding channel and power allocation and starts the training phase for the state value function. The UAV adopts an n-step prediction approach to train both the actor and critic networks. The UAV maintains a trajectory buffer where it stores the collected experience in a first-in-last-out manner. At the beginning of each training session, the UAV records the starting timeslot in $t_{start}$ and then selects an action a(t) to execute. Subsequently, it stores the experience in the trajectory buffer. After reaching step n, the UAV constructs an interaction trajectory in the buffer. The UAV uses the trajectory buffer to update the loss function and cross entropy. The training phase continues until the procedure reaches the convergence point. Then, the critic network is closed, and the actor network makes decisions regarding channel and power allocation.

- Advantages: The protocol is energy-efficient owing to the use of DRL by properly allocating the channel and power.
- Limitations: In the simulation, a dense IoT network was not considered. Additionally, the UAV speed and the localization of the IoT devices were ignored.
- Application domains: Remote hilly and mountainous areas are candidates for this protocol.
- Future improvements: The UAV trajectory should be optimized for efficient data collection within a short time.

#### 5.3.5. DRL-Based Task Scheduling (DRLTS) 

**Figure 15:**
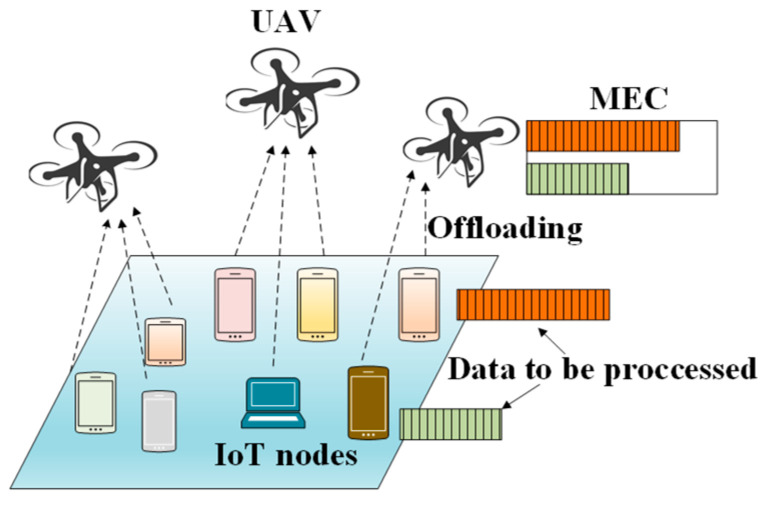
Network architecture of DRLTS.

The authors in [73] proposed a DRL-based task-scheduling mechanism for a UIoT network. Their main objective was to improve the efficiency of task execution for each UAV by using DRL. The IoT devices offload their tasks to nearby UAVs for computation. Figure 15 shows the network architecture of DRLTS where multiple UAV is equipped with MEC and serve many IoT devices for computation offloading. In this protocol, the UAVs function as DRL agents, and each IoT device has several tasks to offload. After the task offloading is complete, the UAVs use DRL to schedule the execution of the tasks. The objective of the DRL algorithm is to avoid slowdowns of the task execution. The current allocated computational resources and the required computational resources for the possible scheduled tasks of the UAV constitute the state space of the DRL algorithm.

The tasks queued by the UAV constitute the action space of the DRL algorithm. The DRL uses a convolutional neural network (CNN) to generate the current *Q*-value, and another CNN to generate target *Q*-value. Furthermore, the loss function of deep *Q*-network (DQN) can be given by

(23) $$L(\theta )=E[(Q^{T}-Q{\left(S_{t},A_{t};\theta \right)}^{2}],$$

where the target function $Q^{T}$ can be formulated as

(24) $$Q^{T}=R_{t}+\gamma \max Q(S_{t+1},A_{t+1};\theta ),$$

where θ is the parameter of the neural network, E represents the offloading level, $Q\left(S_{t},A_{t}\right)$ represents the *Q*-value with state s and action a, $R_{t}$ represents the instant reward on step t, and *γ*(0 < *γ* < 1) represents the discount factor. The action space is divided into sub-steps. In each sub-step, only one sub-action is scheduled. If the task is scheduled in a clustered manner, it will be removed from the waiting queue. After observing the state transition, the agent moves the scheduled task to the appropriate position. If the agent picks up an invalid sub-action, it does not schedule any additional tasks in the current step and instead moves to the next step. The reward is given if the agent minimizes the average slowdown of the task execution. The reward function is given as follows:

(25) $$\sum_{M_{n}}\frac{-1}{t_{z}(u_{n},m_{k})},$$

where $M_{n}$ represents the current set of tasks for the nth UAV, $u_{n}$ represents UAV, $m_{k}$ represents the position of the IoT nodes, $t_{z}$ represents the required execution time.

- Advantages: Load balancing for task offloading is effective owing to the use of the multi-UAV mechanism. DRLTS is effective for minimizing the slowdown of the task-execution process.
- Limitations: In a multi-UAV scenario, communication between UAVs is important for avoiding collisions and achieving proper synchronization. In [63], the communication between the UAVs was not considered.
- Application domains: Remotely deployed MEC-based applications are suitable for this protocol.
- Future improvements: DRL can be used for proper channel selection to minimize the collisions in data transmission.

#### 5.3.6. Space/Aerial-Based Task Scheduling (STS)

The authors of [74] investigated a SAGIN for remote infrastructure-less areas. Their main objective was to efficiently allocate the computational resources to virtual machines (VMs) and schedule the offloaded tasks. After certain tasks are executed locally in each IoT device, the IoT devices offload their tasks to a nearby UAV or to the cloud server through the satellite, to minimize the total system cost for further computation. The offloading decision is made in each timeslot until the IoT device computes some designated tasks locally. However, if a task is offloaded to a UAV, it may not be completed, owing to the mobility of the UAV; in this case, it is not returned to the user at the end of the timeslot. Moreover, in timeslot *t*, multiple tasks can be uploaded to a UAV. In this case, these tasks are executed in different VMs in parallel to reduce the processing latency.

**Figure 16:**
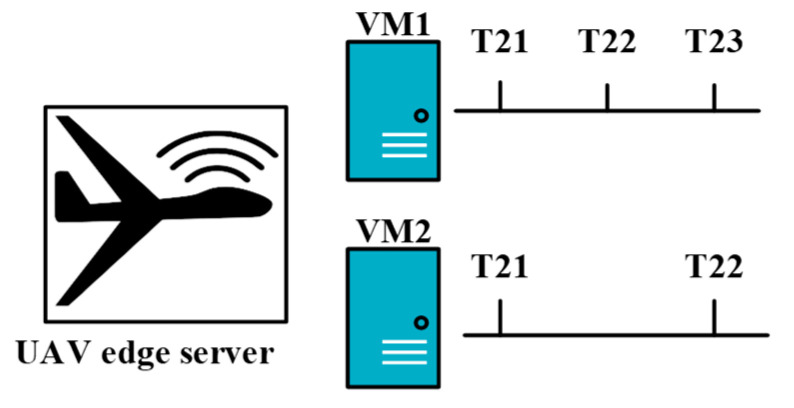
VM allocation and task scheduling of the UAV edge server.

Figure 16 shows that a UAV with an edge server has multiple VMs and it can perform tasks on the VMs concurrently. Some tasks have a delay limit and must be completed at a certain time. To solve this problem, the VM allocation and task scheduling are optimized for reducing the delay in the system. At first, using the MDP computation offloading problem is formulated. The MDP of the STS computing offloading problem is defined as states, actions, transmission probability, and reward. The state of the MDP of STS is defined at the beginning of every time slot. The state includes the path loss information of the current and previous timeslots to learn and predict the path loss information. Actions of MDP refers to the action of scheduling the tasks of the users. The transmission probability of MDP is related to whether the offloaded task to UAV can be completed within the designated time slot or not. The transition probability can be defined as follows:

(26) $$p(s_{t+1}|s_{t},a_{t})=p(PL(t+1)).(T_{r}^{l}(t+1)|T_{r}^{l}(t),a_{t}).p(M(t+1)|M(t),a_{t}),$$

where PL(t) represents the vector of path loss values of all users to their associated UAV, the system state and action space are represented by $s_{t}$ and $a_{t}$, respectively. $T_{r}^{l}\left(t\right)$ represents the remaining time for each user to complete locally processing tasks, and M(t) represents the network state. The reward of the MDP is related to the weighted sum of the delay, energy, and server usage cost. However, due to the rapid mobility and dynamic VM allocation of UAV servers, it is very difficult to model the reward function and the transmission probabilities accurately using the MDP approach. Moreover, when the number of tasks increases, the system becomes intractable. Thus, a DRL-based actor-critic algorithm is adopted for efficient computing offloading. In the actor-critic method, the policy is updated in each timeslot rather than at every episode of task offloading. It helps to reduce the number of samples required to learn the policy.

The learned value function is used as a critic to guide the policy updates at each time slot. Finally, a deep learning method is utilized to learn the policy in terms of policy and state-value function. After applying deep learning, the system state can be observed from the current environment, which is eventually sent to the input of the actor and critic network. Then, the actor network generates actions and updates policy. In each timeslot, for an arbitrary task, there are four possible decisions: not scheduled, process locally, offloads to the edge, and offload to cloud. By mapping these four decisions, the actor network is designed with two output layers. Furthermore, the critic network estimates the value function and updates the parameter. The reward of action is evaluated by the reward evaluator and used to calculate the temporal difference. The temporal difference is used in the update of the policy parameter and the critic network parameter.

- Advantages: The scheme performs well with regard to the average total delay and the total cost of UAV server usage. The UAV server usage is reduced through the DRL-based approach. Moreover, the prioritization of tasks is maintained, enhancing the QoS of the application.
- Limitations: The SAGIN system is expensive and complex to deploy in real application scenarios due to the use of UAVs, a satellite network, and the DRL-based approach [75].
- Application domains: Remote infrastructure-less areas with mobile IoT devices have good application prospects for this protocol.
- Future improvements: Communication between the UAVs and the satellite with proper synchronization should be investigated.

## 6. Comparison of MAC Protocols for UIoT

In this section, the reviewed MAC protocols for UIoT are extensively compared with regard to various aspects. Table 1 presents the key innovative features of the protocols. Table 2 presents the evaluated performance metrics and performance objectives of the discussed protocols. Table 3 presents a comparison of the protocols with regard to various parameters and factors. Researchers and engineers can use these tables to select the most appropriate protocol or design a new MAC protocol. In the following subsections, the performance, unique features, and operational characteristics of the MAC protocols are discussed in detail.

In Table 1, the state-of-the-art MAC protocols for UIoT are summarized with regard to their innovative features, main ideas, and optimization techniques. As shown in the table, the TDMA-based approach is more popular than the other approaches. Limited communication time between UAV and IoT devices may result in significant packet loss. However, this issue can be resolved largely by using the TDMA-based protocol. Different types of optimization techniques are adopted to reach different goals. However, energy consumption minimization is a widely explored area for optimization.

In Table 2, the MAC protocols presented in Section 3 are summarized with regard to their performance metrics. As expected, the protocols that have special mechanisms use more control packets and memory, resulting in additional overhead. However, the special mechanisms are directly related to the performance objectives of the protocols. The effects of the innovative features and performance metrics on the protocols are broadly discussed in Section 6.1.

Table 3 presents a comparison of the reviewed protocols with regard to their parameters and performance metrics. Some of the protocols focus on increasing energy efficiency, whereas others focus on enhancing fairness and scalability. For the protocols designed for remote infrastructure-less areas, the UAVs must offload the computational tasks. The effects of the operational characteristics on protocols are discussed in detail in Section 6.2.

### 6.1. Discussion of Performance and Special Features

In this subsection, we discuss the performance and special features of the reviewed MAC protocols based on Table 1 and Table 2 in detail. The strengths and weaknesses of the special features, as well as the performance criteria, are discussed.

Balanced UAV-IoT [51] is characterized by a tradeoff between throughput and energy consumption. It uses a slotted ALOHA based approach to access a channel by reducing transmission collisions. The main contribution of the protocol lies in the use of two modes of system efficiency and energy efficiency. The protocol can yield high throughput while using system efficiency mode and can use minimum energy during the energy efficiency mode. However, in the performance evaluation of the balanced UAV-IoT system, the multi-UAV scenario is not considered. The performance evaluation is completely focused on varying the density of IoT devices and the flying speed of the UAV. DSC-UAV [57] uses the RAP procedure in a cellular LTE network. It adopts the mechanism of dynamic speed control to maximize data collection efficiency. However, in a dense network, collisions occur when numerous devices attempt to access a channel while the UAV moves at a high speed. In Modified CSMA/CA [60], to maximize the throughput, the CW size is adjusted according to the duration of contact with the UAV for different IoT device clusters. However, the competition among IoT devices in the same cluster for channel access is not considered. The UAV follows a straight trajectory path during data collection. Therefore, the IoT devices that appear first in the trajectory path have the shortest contact duration with the UAV, and the devices that are far from the UAV have the longest contact duration. Thus, the adaptive CW based on the contact duration increases the throughput of the protocol. JRWS [62] uses a basic TDMA based approach for data transmission. It focuses on minimizing the total energy consumption by jointly optimizing the IoT device association, resource allocation, UAV hovering time, WPT duration, and service sequence of the IoT devices. Performance results indicate that the proposed solution outperformed an exhaustive search with regard to the total energy consumption. OTA [63] focuses on the throughput maximization of the whole network. However, there is a tradeoff between throughput and energy consumption. Moreover, the optimal energy consumption for maximizing the throughput is not considered. URS [68] focuses on minimizing the energy consumption of the UAV and IoT devices. The protocol is compared with similar state-of-the-art protocols and outperformed them with regard to energy consumption. However, the delay and throughput of the system are not analyzed in the performance evaluation. UMTAP [65] can resolve multi-frequency tag collisions within a timeslot. However, it cannot prevent collisions between tags with the same frequency in the same timeslot. Typically, in a dense RFID tag-based environment, multiple tags must be identified in the same timeslot. If the tags have different frequency levels, UMTAP can easily differentiate between them and hence prevent collisions. Eventually, this improves the system efficiency.

In SSU [67], the optimal transmission strategy is employed to maximize the number of bits received by the IoT from the cognitive UAV. The UAV keeps the number of slots interfering with the primary user (PU) below a certain threshold, to protect the PU from harmful interference. However, if a spectrum sensing error occurs, the UAV transmits fewer transmission slots and the number of bits received by the IoT devices decreases. SST [23] focuses on securing a trajectory in a military environment using a neural network. WupMDP [70] focuses on avoiding collisions in the wake-up plane to increase the throughput of the system. The performance evaluation indicates that it has a high throughput. However, only one protocol is considered for the comparison, which is insufficient. Moreover, the density of IoT devices and the UAV speed are not considered. DTS [71] adopts a CMDP-based task scheduling mechanism. The performance evaluation results show that the protocol outperformed other mechanisms. The delay for different task arrival rates is also analyzed. DRLCPA [72] utilizes UAV with a DRL-based approach for channel and power allocation to IoT devices for improving the system efficiency. In the performance evaluation, the max–min energy performance is calculated by considering the different trajectory steps of the UAV. The DRL mechanism helps the UAV to learn the change patterns hidden in the environment. The UAV allocates the channel and power to the IoT devices according to the converged DRL.

DRLTS [73] adopts a multi-UAV DRL-based approach to improve the efficiency of the task-execution process for each IoT device. According to the performance evaluation, with an increase in the number of iterations, the total reward converges to an ideal value. This reward value helps the UAV to minimize the average slowdown of the task-execution process and improves the task-execution efficiency. However, an increase in the task arrival rate has a negative effect on the overall system. Therefore, in a very dense network, it is impossible to minimize the slowdown of the task execution. Moreover, in the performance evaluation, the energy consumption of the UAV and IoT devices is not analyzed. STS [74] is a DRL-based computing offloading approach for task scheduling and resource allocation. The performance evaluation results indicate that the total cost of STS is lower than those of greedy and random approaches.

### 6.2. Discussion of Operational Characteristics

In this subsection, we discuss the operational characteristics of the MAC protocols in Table 3 in detail. As indicated by Table 3, all the contention-based MAC protocols [51,57,60] had overhead compared with the contention-free protocols. In the contention-based mechanism, several control packets must be exchanged to establish the connection between the sender and the receiver, which increases the overhead. In contrast, in most of the contention-free MAC protocols [62,63,67,68], control packets do not need to be exchanged if a centralized system schedules the timeslots among the receivers. However, in both cases, the protocol overhead may increase if certain values must be calculated in each slot or iteration. This also results in a shortage of memory space. For example, in Modified CSMA/CA [60], the CW size must be calculated for each cluster. In contrast, in the UMTAP [65], the numbers of successful slots, collided slots, and empty slots must be calculated at the end of each slot. Therefore, this protocol produces overhead. Moreover, in most of the AI-based algorithms [23,70,71,72,73,74], certain values must be stored in each iteration and used later to make decisions. However, in the learning phase, the overheads increase, but after the algorithm converges to the optimal point, the overheads start to decrease.

As shown in Table 3, all the AI-based protocols are significantly more adaptive and scalable than the conventional contention-based and contention-free mechanisms. This is because AI-based protocols take advantage of interactions with the environment; they learn from the environment and make appropriate decisions based on the experience.

With regard to fairness, contention-free mechanisms outperform contention-based mechanisms. Contention-free mechanisms allocate timeslots for all the existing IoT devices to transmit data; hence, the fairness is high. Moreover, in contention-free mechanisms, there is no packet loss and no collisions between data. The fairness is also high for DRL-based mechanisms because DRL is applied on top of the contention-free mechanism.

Energy efficiency is one of the crucial factors for UIoT networks because most of the devices are energy-constrained. However, as indicated by Table 3, few protocols addressed this issue. Therefore, further research in this area is necessary.

Load balancing occurs when multiple UAVs are deployed in the system. Although the UAV power is rechargeable, it depends on the battery power during UAV flight. Moreover, the UAV has a limited computation capability. It is difficult for a single UAV to perform all the computational tasks offloaded by the IoT devices. Therefore, deploying only a single UAV makes the system unreliable and vulnerable. When multiple UAVs are deployed, they can perform load sharing and balancing, enhancing the overall system performance.

It can be observed from Table 3 that each of the protocols considers different values for UAV transmission range, UAV velocity, UAV altitude, data rate, and bandwidth. However, the widely adopted UAV transmission range and UAV altitudes are between 100–500 m and 5–100 m, respectively. Data rate and bandwidth vary from application to application. Very little similarity in data rate and bandwidth value is observed.

### 6.3. Lessons Learned

From the above-mentioned discussions, we can conclude that it is not practical to define the most suitable MAC protocol for UIoT in a generalized way. The most suitable MAC protocol for UIoT is application- and requirements-specific. However, the widely adopted MAC protocol, CSMA/CA, could be a good choice in UIoT network due to its flexible access strategy. On the other hand, the TDMA scheme may not be an effective solution to deal with the heterogeneity of the access time. If the location of each IoT device is known by the UAV prior to data collection through a control center, the trajectory can be planned to serve those IoT devices. However, a hybrid MAC protocol can be a better choice for MAC protocol of UIoT because it can enjoy the benefits of both contention-based and contention-free approaches. Furthermore, an AI-based approach is suitable for the applications that need decision making such as computation offloading and task scheduling.

## 7. Open Issues and Research Challenges of MAC Protocols for UIoT

UIoT MAC protocols are still in the development phase. Hence, an energy-efficient, robust, and flexible MAC protocol needs to be developed. In this section, issues for future research are discussed, most of which present significant challenges. We have also provided recommended solutions for each of the issues.

### 7.1. Energy Consumption and Network Lifetime

Most IoT devices and cost-effective drones are battery-powered and can remain powered for a limited period. Hence, for a long network lifetime, efficient energy consumption is essential for both UAVs and IoT devices. Although numerous studies have been performed on the energy efficiency of IoT networks [76,77], several challenges must be overcome for reducing energy consumption such as overhearing, collisions, and idle listening. Using solar power is an option for energy harvesting from natural resources. However, on rainy days and during the winter season, solar power is typically unavailable. Moreover, when a collision occurs, packets need to be retransmitted. A large amount of energy is wasted owing to this problem.

Recommended solutions: Dynamic sleep and wake-up based duty cycling mechanism can help to reduce unnecessary energy wastage in monitoring applications. However, reducing the number of transmissions, proper handling of collisions, and solving the hidden and exposed nodes problem can reduce energy consumption and enhance the network’s lifetime.

### 7.2. Privacy and Security

Privacy and security are also major concerns for heterogeneous networks because both UAVs and IoT devices can operate in sensitive, unguarded, and remote areas that are prone to third-party attacks. Moreover, UIoT networks are likely to encounter numerous challenges because of their dependence on wireless communication. If this technology is used with malicious intent, the implications can be detrimental to society. For example, if terrorists attack a surveillance UAV, they can steal all the information regarding places or people in real-time, which can be life-threatening.

Recommended solutions: One of the promising technologies to handle the security-related issues in communication is the integration of blockchain with UIoT. Blockchain technology is a highly disruptive technology that was first mentioned by Satoshi Nakamoto in 2008 via the usage and benefits of Bitcoins [78]. Blockchain technology can enable UIoT with cryptographic techniques to ensure secure communication. This feature of the blockchain makes it suitable for such applications that contain critical information such as defense, healthcare, and financial applications. Very few studies have been carried out which focus on the blockchain in UIoT communication. One of the related studies is [79] which proposed a blockchain-based secure data acquisition system from IoT devices using UAV. 

Another approach to secure UIoT system is to use different machine learning mechanisms, especially deep learning with neural network techniques. It is because the deep learning-based approaches can train itself by interacting with the environment via trial and error. Thus, it can avoid future attacks by learning from their experiences. One such kind of related work is presented in [80]. However, further studies are needed to provide counter measurements for faulty nodes, erroneous communication channels, spoofing, and denial-of-service attacks. To protect networks from these attacks, cryptographic mechanisms with blockchain such as data encryption and hashing techniques should be adopted. Additionally, handshaking techniques, as well as the sending and receiving of acknowledgments, are effective options for secure communication.

### 7.3. Interoperability

Interoperability is concerned with the ability of different systems, devices, applications, or products to connect and communicate in a coordinated way without any effort from the end-users. Functions related to interoperability includes data access, data transmission, and cross-organizational collaboration regardless of its developer or origin. In a UIoT system, interoperability is a major concern because different systems (such as UAV and IoT devices) with different characteristics try to connect and communicate with each other. The interoperability among the devices should be smooth enough so that they can effortlessly achieve their targeted goals. 

Recommended solutions: In most of the UIoT systems, there exists a system orchestrator or central controller which ensures the interoperability among the different devices [81]. For example, the system orchestrator instructs the UAV on how, where, and which access technology (such as FANET, cellular, ad-hoc, etc.) to use to collect and deliver the necessary information. In case of disaster and emergency situations where multiple UAVs and IoT devices are used, the UAVs using the cellular technology can be used in the coverage areas and the UAVs using satellite connectivity can operate in the infrastructure-less areas. However, further investigation is needed to ensure interoperability among the devices in an efficient and uninterrupted manner.

### 7.4. Integration of Machine Learning

In recent years, machine learning has become an important technology in the UAV and IoT industries. Machine learning techniques help the devices to take context-based decisions and learn from their experiences. This leads to the creation of the term “Cognitive UIoT” [82]. Several studies have been carried out regarding the integration of machine learning mechanism with IoT and UAV. The authors in [83] used a supervised learning-based machine learning mechanism to prevent road accidents by creating a framework to detect the driver’s consciousness. However, in the UIoT, one of the open problems is the reliable extraction of real-world IoT data from a noisy, complex, and remote environment that confuses conventional machine learning mechanisms. Therefore, more research works need to be conducted to address this issue for reliable information collection and processing.

Recommended solutions: Edge computing moves more computational power and resources closer to end-users, by increasing the number of endpoints and locating them nearer to the consumers. Edge computing can be utilized to satisfy the demands of reliable extraction of IoT data from remote environments, and machine learning can be applied to feature extraction from the data sources.

### 7.5. Scalability and Large Network Size

Similar to most wireless-sensor and ad-hoc networks, a UIoT network depends primarily on multi-hop and peer-to-peer communications without centralized control. MAC protocols should not impose undesirable computational weights on a system, which would necessitate excessive memory for preserving state information. A hybrid scalable MAC protocol for IoT networks was designed in [84] which can accommodate a large number of IoT devices without much computational overhead. However, due to the unpredicted topology changes and dynamic characteristics of UIoT, the MAC protocols targeted for IoT networks are not suitable for UIoT.

Recommended solutions: Efficient clustering of IoT devices can reduce the number of transmissions from IoT devices to UAVs. Moreover, considering the mobility of the UAV and the limited communication time with IoT devices, the prioritized access to the channel can enhance the fairness and scalability of the overall system.

### 7.6. Congestion Control

In a heterogeneous UIoT network, numerous devices and sensors in the coverage area attempt to access a shared medium simultaneously, which can result in network congestion and collision. Eventually, the system performance is degraded, and a large number of packets are dropped. Some studies related to congestion control have been investigated in the literature. A congestion-control mechanism employing a rate-based approach for the bursty traffic of IoT applications is presented in [85]. However, research works related to the congestion control mechanism for UIoT networks are still not presented in the literature. 

Recommended solutions: Depending on the application types, different mechanisms can be used to tackle the congestion. For small-packet event-based applications, the phase shifting of IoT nodes can be the best choice to control congestion. On the other hand, in periodic and continuous applications, an efficient transmission scheduling mechanism by avoiding collisions seems to be a promising solution due to the huge number of packets. Additionally, to tackle the congestion control in UIoT, some suggestions can be considered as in a priority access scheme [86]. On the other hand, applying an open-loop congestion-control mechanism before starting data transmission can prevent network congestion [87].

### 7.7. Cross-Layer Architecture

In a heterogeneous IoT network, successful data transmission between different types of devices is important for network reliability. In this context, the cross-layer design of protocols attracts increasing attention. Cross-layer protocols can improve energy consumption and bandwidth significantly. The authors of [88] designed a cross-layer MAC protocol using template metaprogramming techniques. However, challenges must be addressed for designing effective cross-layer protocols. The side effects of unforeseen dependencies among the layers, instability, and protocol overhead should be investigated. Therefore, frameworks that can overcome these challenges and provide reliability, flexibility, robustness, and interoperability are necessary.

Recommended solutions: A universal solution for cross-layer architecture problems might be non-existent and even not practical. However, the applications that share common functionalities can be used to develop a universal design for specific applications. For example, different video streaming applications have different cross-layer design implementations. The implementations can be studied extensively and applied to develop an efficient mechanism for UIoT applications with video streaming.

### 7.8. Performance Evaluation Tools

Numerous simulation tools have been used for the MAC protocols of UIoT, such as MATLAB and NS-3. However, no dedicated simulation tools for developing MAC protocols for UIoT networks are currently used. In most cases, researchers simulate UIoT MAC using a simulator that is dedicated to a homogeneous wireless-sensor network. Simulating a heterogeneous network is highly challenging. Therefore, the validation of currently available simulation tools is challenging.

Recommended solutions: To validate and obtain the proper performance results of various protocols, it is recommended to experiment with the proposed algorithm with the most widely used network simulator as well as with real testbed scenarios.

## 8. Conclusions

In this article, the existing MAC protocols designed for UIoT networks were investigated in detail and comparatively analyzed. The communication architecture and various design considerations for UIoT networks were briefly introduced. The MAC protocols were classified into three types: contention-based, contention-free, and AI-based. Subsequently, we discussed the main objective and innovative features, operational principles and advantages, limitations, and potential future improvements of the existing MAC protocols of UIoT. The existing MAC protocols were extensively compared with regard to their key characteristics, performance metrics, and operational characteristics. Finally, the open issues and research challenges were summarized and recommended solutions for each open issue are provided.

The key achievements of this work are as follows: Based on the study, we can see that most of the MAC protocols for UIoT cover partial objectives rather than covering the entire MAC aspects. For example, the protocol suggested for throughput does not consider delay and energy performance. The AI-based solutions do not consider UAV’s mobility and communication duration of time. Multi-UAV development can be observed in some research works, in which computation offloading is needed. UAV’s energy is one of the bottlenecks of the whole architecture. Though the remaining energy and lifetime of UAV are essential to make a practical solution, very few protocols have considered them. To the best of our knowledge, no survey covers the MAC protocols dedicated for UIoT. Hence, this paper will provide an overall idea of this very recent and emerging topic. This paper has been written to facilitate the new researchers with an in-depth knowledge of the topic, by criticizing the papers intensely.

According to the results of our survey, both the high node density and high mobility of UAVs should be considered in all MAC protocols for UIoT systems. As a future work, we will propose our own MAC protocol considering the dynamicity and heterogeneity of the UIoT networks. Finally, we believe that the presented information will serve as a beginning for research on MAC protocols for UIoT and will also create research interest in this field.

## Figures and Tables

**Table 1:** Summary of MAC protocols for UIoT with regard to main ideas, innovative features, and optimization techniques.

Protocol	Year	Main Ideas	Innovative Features	Optimization Techniques
Balanced UAV-IoT [51]	2019	Slotted ALOHA-based approach is adopted.	Two modes are adopted: energy-efficient mode and system efficiency mode.	Locating optimal parameters utilizing the particle swarm optimization algorithm.
DSC-UAV [57]	2018	Physical RAP is adopted under LTE/LTE-A network.	UAVs can adaptively adjust their speed to maximize data-collection efficiency.	–
Modified CSMA/CA [60]	2020	CSMA/CA-based approach is adopted.	CW size is adjusted according to the contact duration between UAV and IoT device.	–
JRWS [62]	2019	TDMA-based workflow model is adopted for data transmission and computation.	Different optimization techniques are used.	UAV energy minimization problem is solved by the block coordinate descent method. The resource allocation problem is solved by the Lagrange dual method. The optimal IoTD sequence is obtained using flow shop scheduling techniques.
OTA [63]	2019	Dynamic TDMA-based frame structure is adopted.	UAV is equipped with a full-duplex HAP.	Optimal time allocation strategy is solved by Karush–Kuhn–Tucker conditions.
UMTAP [65]	2019	TDMA-based approach is adopted.	Multi frequency tag collision is resolved.	Optimal frame length is calculated using Limit theorems and Taylor’s formula.
SSU [67]	2019	TDMA-based approach is adopted.	Optimal transmission strategy is used, with the number of transmission slots below a threshold level.	Successive convex approximation algorithm is used to optimize imperfect spectrum sensing.
URS [68]	2019	TDMA-based approach is adopted.	Each IoT device can simultaneously perform offloading and local computation.	Computation resource scheduling, bandwidth allocation, and UAV’s trajectory are optimized using successive convex approximation methods.
SST [23]	2019	ANN-based security framework is adopted.	Three types of communication links are used: UAV-to-UAV, UAV-to-device, and virtual.	–
WupMDP [70]	2017	MDP-based approach is adopted.	MDP comprises five stages: successful transmission, collision, Idle1, Idle2, and Idle3.	–
DTS [71]	2019	MDP-based approach is adopted.	UAV is equipped with two communication interfaces.	Energy consumption is optimized using constrained MDP.
DRLCPA [72]	2019	Deep learning-based approach is adopted.	UAV-BS uses a trajectory buffer to store learned experiences in a first-in-last-out manner.	The DRL-based actor-critic network is used to optimize energy consumption and enhance system performance.
DRLTS [73]	2020	Deep learning-based approach is adopted.	Reward function directs the agent to minimize the average slowdown for UAVs.	An approximation algorithm is used to determine all the connections between UAV and IoT devices.
STS [74]	2019	Deep learning-based approach is adopted.	UAV employs different VMs for different tasks.	DRL is used to find the optimal offloading policy.

**Table 2:** Summary of MAC protocols for UIoT with regard to performance metrics and objectives.

Protocol	Reference	Evaluated Performance Metrics	Performance Objective
Balanced UAV-IoT	[51]	Number of competing tags in each reading cycle, maximal allowable flying speed, energy-efficiency gain	Maximize throughput with minimum energy consumption
DSC-UAV	[57]	Successful access probability, average access delay, block probability, data-collection efficiency, collision probability	Maximize data-collection efficiency
Modified CSMA/CA	[60]	Saturation throughput for different device densities, retry limit, CW size, radius	Increase saturation throughput
JRWS	[62]	Energy consumption, weighted energy consumption, total hovering time	Minimize total energy consumption
OTA	[63]	Total throughput	Maximize total throughput
UMTAP	[65]	System efficiency, tag identification time	Increase system efficiency
SSU	[67]	Average data rate, average number of bits	Maximize number of bits received by IoT devices
URS	[68]	Total task completion time, weight for energy consumption of UAV, uniform size ratio of task-output data to task-input data, separate energy consumption for IoT devices and UAV, weighted sum of energy consumption for UAV and IoT devices	Minimize weighted sum of energy consumption of UAV and IoT devices
SST	[23]	Macaulay duration for network resources, link depletion at different values of variables, link duration based on Macaulay-duration conditions, network learning rate	Secure link connectivity
WupMDP	[70]	Number of successfully transmitted packets, gain between successfully transmitted and collided packets	Increase number of successfully transmitted packets
DTS	[71]	System delay vs. energy consumption, system drop rate, and task arrival rate	Reduce delay
DRLCPA	[72]	Moving-average max–min energy efficiency of IoT nodes with varying number of nodes, moving-average max–min energy efficiency of IoT nodes with different algorithms	Maximize minimum energy efficiency among all IoT nodes
DRLTS	[73]	Reward, average slowdown, fitness	Improve task-execution efficiency for each UAV
STS	[74]	Average total delay vs. UAV edge server computational resources, total number of tasks, total cost	Reduce total cost

**Table 3:** Comparison of MAC protocols with regard to operational characteristics.

Reference	MAC Type	Protocol Overhead	UAV Transmission Range	Scalability	Fairness	Energy Efficiency	Load Balancing	UAV Speed (m/s)	UAV Altitude (m)	Number of UAVs	Data Rate	Bandwidth
[51]	C	Y	150–550	H	Me	Y	N	0–120	0–300	S	40 kbps	-
[57]	C	Y	30	L	Me	N	N	0–100	-	S	-	-
[60]	C	Y	1000	L	Me	N	N	10–20	-	S	1 Mbits	-
[62]	CF	N	-	H	H	Y	N	-	5	S	-	10 MHz
[63]	CF	N	-	L	H	N	N	10	5, 10	S	-	-
[68]	CF	N	-	L	H	Y	N	-	10	S	-	30 MHz
[65]	CF	Y	200	H	H	N	Y	-	-	Mu	2 Mbps	-
[67]	CF	N	L	L	H	N	N	15	50, 80	S	-	2 MHz
[23]	NN	Y	-	L	H	N	Y	11.11	-	Mu	-	-
[70]	MDP	Y	-	L	H	N	Y	-	-	Mu	-	-
[71]	MDP	Y	500	H	H	N	N	-	100	S	-	10 MHz
[72]	DRL	Y	40	H	H	Y	N	-	100	S	-	1 kHz
[73]	DRL	Y	100	H	H	N	Y	-	100	Mu	-	1 MHz
[74]	DRL	Y	-	H	H	Y	Y	-	90	Mu	10 Mbps	20 MHz

Note: “-” indicates that the data were not provided in the corresponding literature, “Y” represents “Yes”, “N” represents “No”, “H” represents “High”, “L” represents “Low”, “Me” represents “Medium”, “S” represents “Single”, “Mu” represents “Multiple”, “C” represents “Contention-based”, “CF” represents “Contention-free”, “NN” represents “Neural network-based”, “MDP” represents “Markov decision process-based” and “DRL” represents “Deep reinforcement learning-based”.
